# A tumor-targeted heptamethine cyanine dye induces suppression of progesterone receptor activity to treat hormone receptor-positive breast cancer

**DOI:** 10.7150/thno.126396

**Published:** 2026-01-01

**Authors:** Yoonbin Park, Sang-Hyo Kim, Moon Suk Kim, Hoon Hyun

**Affiliations:** 1 Department of Biomedical Sciences, Chonnam National University Medical School, Hwasun 58128, South Korea.; 2 BioMedical Sciences Graduate Program (BMSGP), Chonnam National University, Hwasun 58128, South Korea.; 3 Department of Molecular Science and Technology, Ajou University, Suwon 16499, South Korea.

**Keywords:** breast cancer, progesterone receptor, heptamethine cyanine dyes, immunogenic cell death, tumor targeting

## Abstract

**Background:** A primary treatment of hormone receptor-positive breast cancer is the pharmacological inhibition of hormone receptors by blocking the effects of estrogen and/or progesterone.

**Methods:** In MCF-7 xenograft tumors known as estrogen-sensitive breast cancer, a hydrophilic near-infrared (NIR) heptamethine cyanine dye (named CA800-PR) induced Golgi fragmentation and suppressed only progesterone receptor protein expression, regardless of estrogen receptors.

**Results:** In this study, CA800-PR was newly developed for the treatment of hormone receptor-positive breast cancer unlike conventional drugs such as tamoxifen and aromatase inhibitors. Since the intracellular stress induced by CA800-PR led to the production of pro-inflammatory cytokines, we confirmed a significant increase in the presence of antitumor/pro-inflammatory MHC class II^+^ CD80^+^ M1-type macrophages during the course of treatment. Comparing with the traditional hormone therapies aimed at controlling tumor growth or preventing recurrence post-surgery, the tumor-targeted NIR fluorescent dye CA800-PR alone can be effectively used as a multifunctional antitumor agent directly inducing apoptosis in both MCF-7 cells and xenograft tumors.

**Conclusion:** This work provides a promising alternative to hormone therapy-related breast cancer for future clinical applications.

## Introduction

Breast cancer is commonly classified into four major clinical subtypes according to the presence or absence of molecular markers for estrogen receptor (ESR), progesterone receptor (PGR), and human epidermal growth factor receptor 2 (HER2). Hormone receptor-positive (HR^+^) breast cancer is the predominant subtype with a favorable prognosis, accounting for approximately 70-80% of all diagnosed breast cancers, expressing at least one type of hormone receptor (ESR and PGR) 1-3. These receptors serve as fundamental molecular markers that are well-recognized and established prognostic factors, as well as predictors of therapeutic response in clinical practice. Hormone therapies, primarily tamoxifen and aromatase inhibitors, have significantly improved prognosis and survival rates in HR^+^ breast cancers, yet 30% of patients have primary resistance to the therapy, due to acquired ESR1 mutations, and 40% of them eventually face high-risk situations for relapse 4,5. In this respect, hormone therapy alone is insufficient to prevent breast cancer relapse. Thus, recent efforts have focused on novel therapeutic strategies including new endocrine drugs, combination therapies, and immunotherapies to prevent resistance and relapse in HR^+^ breast cancer treatment.

Although the most common primary treatment for HR^+^ breast cancers have come from targeting ESR signaling, there has recently been renewed interest in utilizing the new synthetic PGR-specific ligands, such as mifepristone (commonly known as RU486), norethindrone, and mometasone furoate, for breast cancer management 6,7. Among them, mifepristone is a potent synthetic steroid acting as a PGR antagonist associated with anti-progestational effects, which can influence progesterone's role in breast carcinogenesis 8. Especially, previous studies have demonstrated that mifepristone promotes remission of tumor growth, when combined with an antiestrogenic drug like tamoxifen, and induces apoptosis in antiestrogen-resistant breast cancer cells such as MCF-7 and MDA-231 cells 9,10. Since it is known that PGR can synergize with or antagonize ESR to influence downstream biological processes 11, it is important to develop the new type of PGR-specific small molecules for a simple and more effective treatment of HR^+^ breast cancers without the need of combination treatments.

**Scheme 1:**
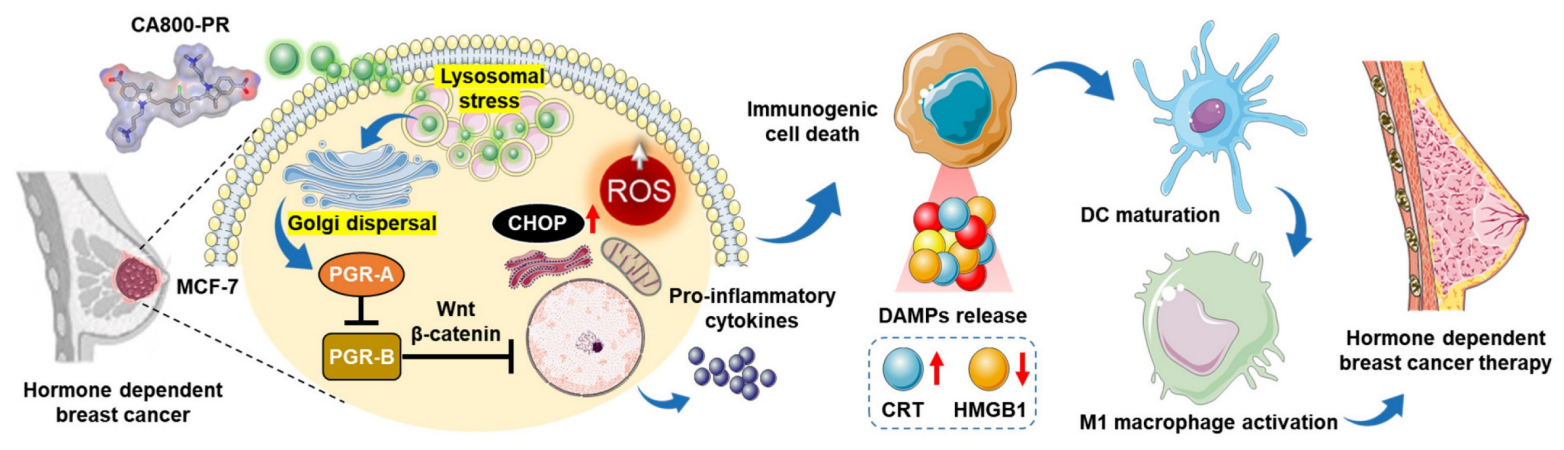
Schematic illustration of the action mechanism of CA800-PR. After localized in lysosomes, CA800-PR induces Golgi dispersal leading to the suppression of PGR activity by downregulating the PGR-A isoform. CA800-PR also increases the levels of intracellular ROS and CHOP protein, contributing to the process of immunogenic cell death to treat hormone receptor-positive breast cancer.

Over the last decade, the concept of “structure-inherent cancer targeting” using single small molecule NIR dyes has been studied to achieve simultaneous cancer-specific targeting, imaging, and therapy in cancer theranostics. Since the NIR dye itself unexpectedly shows potent therapeutic efficacy in certain types of cancer, polymethine cyanine-based functional dyes have been continuously developed to apply in various types of cancer through this strategy without further chemical conjugation to tumor targeting ligands or therapeutic drugs 12,13. In this study, we newly designed a water-soluble zwitterionic heptamethine cyanine dye, named CA800-PR, to enable tumor-specific uptake, targeted NIR fluorescence imaging, and enhanced therapeutic efficacy in the HR^+^ breast cancer. For the first time, we report that the NIR fluorescent small molecule CA800-PR not only blocked the PGR but also triggered generation of reactive oxygen species (ROS) through Golgi fragmentation. Consequently, the intracellular stress could lead to the production of pro-inflammatory cytokines, resulting in the induction of immunogenic cell death (ICD). As a result of the activation of an immunomodulatory response, we confirmed a significant increase in the presence of antitumor/pro-inflammatory MHC class II^+^ CD80^+^ M1-type macrophages during the course of treatment (Scheme 1). Overall, this work may offer a promising strategy to overcome the endocrine resistance and improve treatment outcomes in the HR^+^ breast cancer.

## Results and Discussion

### Synthesis, characterization, and *in vitro* assay of CA800-PR

**Figure 1:**
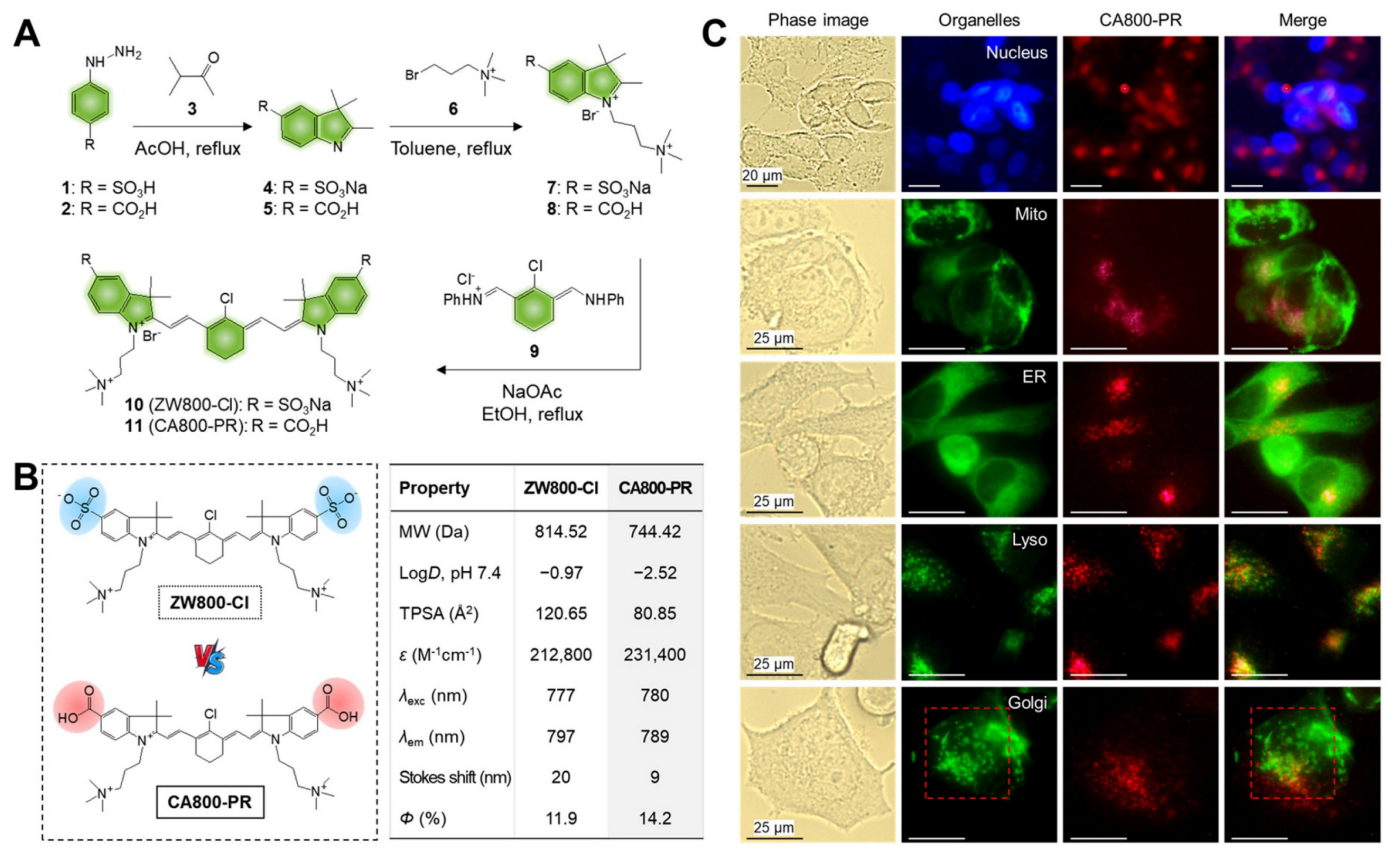
Synthesis and characterization of ZW800-Cl and CA800-PR. A) Synthetic scheme of the water-soluble heptamethine cyanine dyes ZW800-Cl and CA800-PR. B) Chemical structures and physicochemical/optical properties of ZW800-Cl and CA800-PR. *In silico* calculations of the distribution coefficient (log*D* at pH 7.4) and topological polar surface area (TPSA) were calculated using Marvin and JChem calculator plugins (ChemAxon). C) Live-cell imaging of CA800-PR in MCF-7 cells. CA800-PR was co-stained with DAPI, Mito-, ER-, Lyso-, and Golgi-Trackers, respectively. Phase contrast and fluorescence images are obtained after 24 h of incubation with 100 μM of CA800-PR, followed by staining with the commercial organelle trackers. Golgi fragmentation is indicated by the red dotted rectangle. Images are representative of *n* = 6 independent experiments. All fluorescence images had identical exposure times and normalization. Scale bars = 25 μm.

The water-soluble NIR small molecule dye CA800-PR was developed based on the zwitterionic heptamethine cyanine dye, named ZW800-Cl, reported previously 14. It is well-known that the zwitterionic polymethine dyes show not only superior optical and physicochemical properties including high molar extinction coefficients, high water solubility, and good water stability, but also excellent *in vivo* performances such as low nonspecific tissue/organ uptake and fast renal excretion from the body 15. Thus, CA800-PR was newly designed and synthesized through a condensation reaction in which two carboxyl- and two trimethylammonium groups combine with a heptamethine bridge containing meso-chloride (Figure 1A). The structure determination and exact molecular weights of ZW800-Cl and CA800-PR were confirmed by ^1^H NMR and mass spectrometry coupled with chromatography to validate their successful synthesis for the next *in vitro* and *in vivo* experiments (Figures S1-2). After purification, the absorption and fluorescence emission spectra of ZW800-Cl and CA800-PR were measured in phosphate-buffered saline (PBS, pH 7.4) (Figure S3). As summarized in Figure 1B, CA800-PR displayed the higher extinction coefficient (231,400 M^-1^cm^-1^) and fluorescence quantum yield (14.2%), as compared with that of the representative zwitterionic dye ZW800-Cl (212,800 M^-1^cm^-1^ and 11.9%, respectively). Next, *in silico* prediction of the physicochemical characteristics, such as hydrophobicity and chemical polarity, was performed using JChem software. The carboxylated CA800-PR has a relatively lower hydrophobicity (log*D* = -2.52) and polarity (TPSA = 80.85 Å^2^), comparing with that of the sulfonated ZW800-Cl (-0.97 and 120.65 Å^2^, respectively).

To investigate the cell type-specific toxicity of CA800-PR, we examined the cell viability and observed the intracellular uptake using different types of human cancer cells, including HT-29, NCI-H460, MDA-MB-231, MCF-7, and T47D, as well as noncancerous NIH/3T3 fibroblasts and MCF-10A normal breast epithelial cells. Based on the results of the 3-(4,5-dimethylthiazol-2-yl)-2,5-diphenyltetrazolium bromide (MTT) assay, CA800-PR showed no significant cytotoxicity to the HT-29, NCI-H460, and MDA-MB-231 cancer cells even at the high concentration of 100 μM. As expected, the noncancerous NIH/3T3 fibroblasts and MCF-10A cells also exhibited no cytotoxic effects between 2 and 100 μM concentrations of CA800-PR, exactly similar to that of the cancer cells (Figure S4A). These results confirm that the zwitterionic polymethine dyes do not interact with cell membranes, consistent with previous studies 16. Importantly, the viability of MCF-7 and T47D breast cancer cells after treatment of CA800-PR decreased relatively with an increase in concentration. This result demonstrates that CA800-PR has cell type-specific toxicity and can be used for targeted imaging and treatment of HR^+^ breast cancer. After confirming the cytotoxic effect of CA800-PR on MCF-7 cells, we observed the intracellular uptake of CA800-PR after 24 h of incubation in MDA-MB-231 and MCF-7 cells. Interestingly, CA800-PR displayed low NIR fluorescence signals in the MDA-MB-231 cells, whereas CA800-PR revealed distinct intracellular localization with strong fluorescence signals in the MCF-7 cells (Figure S4B). However, the internalization mechanism remains unclear, and there remains a question of whether the carboxyl groups of CA800-PR may affect the intracellular uptake compared with that of the sulfonated zwitterionic polymethine dyes 17.

Additionally, we further explored the intracellular localization of CA800-PR to determine whether CA800-PR may co-localize with the major organelles including nucleus, mitochondria (Mito), endoplasmic reticulum (ER), lysosome, and Golgi apparatus. CA800-PR was co-stained with DAPI, Mito-, ER-, Lyso-, and Golgi-Trackers, respectively, to identify the locations of CA800-PR compared with major organelles after internalization (Figure 1C). Significantly, CA800-PR was co-localized with the Lyso-Tracker compared with that of other Trackers. It is known that lysosomal stress can initiate Golgi stress, and in turn, Golgi stress can further exacerbate lysosomal dysfunction, creating a feedback loop or “vicious cycle”. More importantly, we confirmed that the green-fluorescent Golgi-Tracker was stained with Golgi fragmentation, unlike normal Golgi morphology, after 24 h of incubation with 100 μM of CA800-PR. To reconfirm whether the Golgi fragmentation was induced by CA800-PR, we observed the time-dependent changes in the Golgi apparatus using a confocal microscope during 24 h of incubation after treatment of CA800-PR (Figure S5). Significantly, the normal Golgi morphology stained with the green-fluorescent Golgi-Tracker started to spread throughout the cytoplasm after 4 h of incubation, and we clearly observed the Golgi dispersal in the entire complete cytoplasmic region at 8 and 24 h post-incubation. This indicates that CA800-PR located in the lysosomes could contribute to induction of Golgi fragmentation and dysfunction, which can be related to the MCF-7-specific apoptosis. Thus, the Golgi fragmentation plays a role in the cell death pathway with implications for immune responses 18.

### *In vivo* tumor targeting and antitumor efficacy of CA800-PR

**Figure 2:**
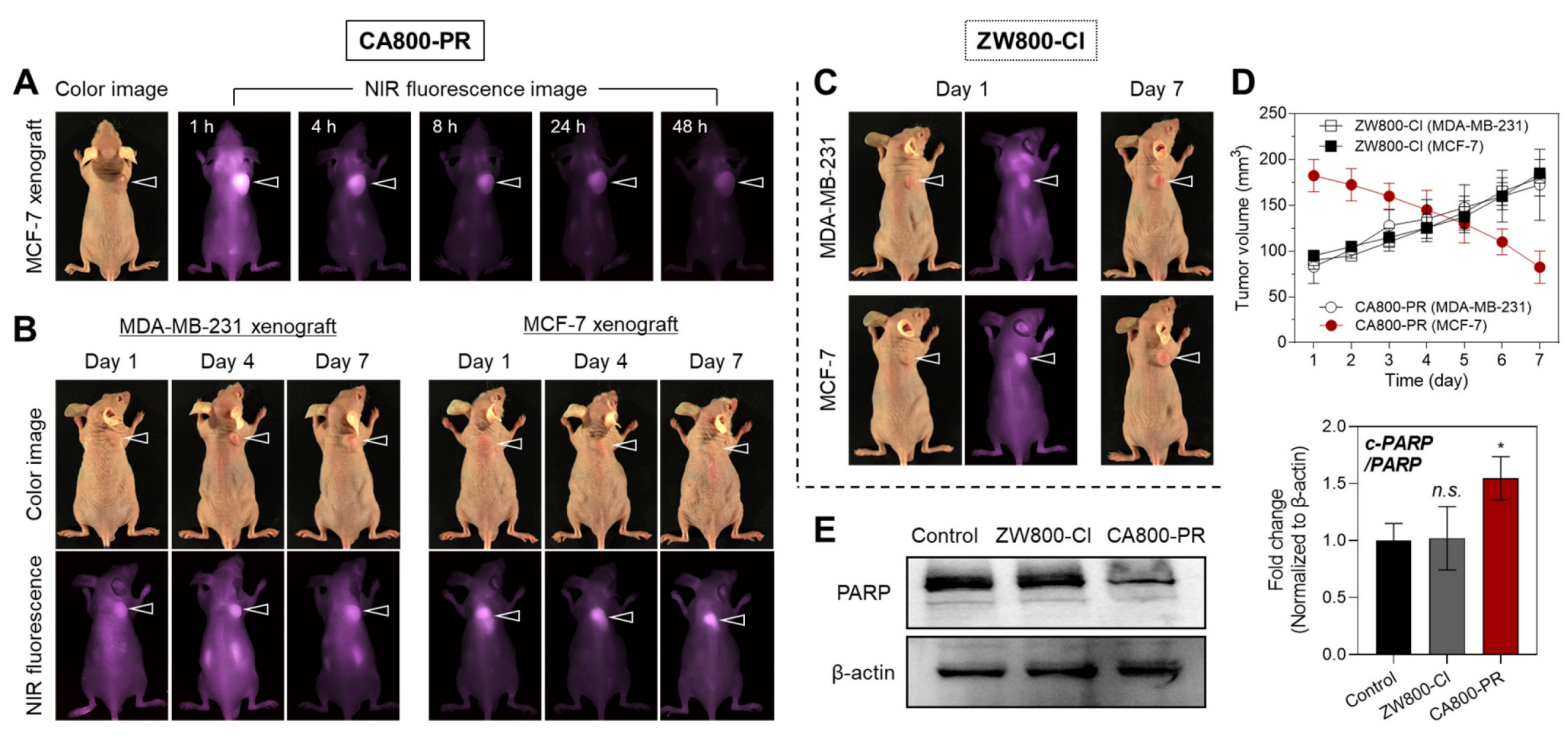
*In vivo* tumor targeting efficiency and therapeutic effect of CA800-PR. A) Time-dependent NIR fluorescence imaging for 48 h after injection of CA800-PR. MCF-7 tumor-bearing mice were intravenously injected with CA800-PR (1.5 mg kg^-1^, *n* = 6). The tumor site is indicated by an arrowhead. B) Antitumor effect of CA800-PR in MDA-MB-231 and MCF-7 xenograft mouse models. Tumor-bearing mice were intravenously injected with CA800-PR (1.5 mg kg^-1^, *n* = 6) every 2 days (a total of 4 times in 7 days). The tumor site is indicated by an arrowhead. C) Antitumor effect of ZW800-Cl in MDA-MB-231 and MCF-7 xenograft mouse models. Tumor-bearing mice were intravenously injected with ZW800-Cl (1.6 mg kg^-1^, *n* = 6) every 2 days (a total of 4 times in 7 days). The tumor site is indicated by an arrowhead. All NIR fluorescence images have identical exposure times and normalization. D) Tumor growth rates of each injection group were monitored for 7 days (*n* = 6). E) Western blot and quantitative analysis of PARP cleavage as an apoptosis marker in the MCF-7 xenograft model. *β*-actin was taken as the loading control. Data are expressed as mean ± S.D. (^*^p < 0.05, *n* = 3).

As shown in Figure 2A, the *in vivo* tumor-targeting efficiency of CA800-PR was evaluated in MCF-7 xenograft mice after intravenous administration. *In vivo* NIR fluorescence imaging in real-time was performed to confirm the tumor-specific accumulation and retention of CA800-PR until 48 h of injection. Remarkably, CA800-PR revealed excellent tumor targeting ability *in vivo* and the strong fluorescence signal in the tumor site gradually decreased for 48 h of injection. Meanwhile, the biodistribution and clearance of CA800-PR in the major organs was examined by *ex vivo* fluorescence imaging of organs and tissues harvested at 4 and 24 h post-injection (Figure S6A,B). As expected, the most of CA800-PR was eliminated from the body within 24 h of administration. This indicates that the zwitterionic surface charge of CA800-PR improve the biodistribution and clearance to prevent nonspecific organs/tissues uptake. Also, the pharmacokinetic profile of CA800-PR was evaluated after intravenous administration (Figure S6C). The elimination half-life (*t*_1/2*β*_) was estimated to be 21.01 min, which is comparable to that of ZW800-Cl (28.08 min) reported previously 14.

Based on the concept of “structure-inherent cancer targeting”, there are two well-known theories to explain the tumor targeting mechanism for the heptamethine cyanine dyes containing a chloro-cyclohexenyl ring. Firstly, organic anion transporting polypeptides (OATPs)-mediated uptake of the heptamethine cyanine dyes into cancer cells has been the predominant mechanism by which OATP transporters are well recognized to be overexpressed in a variety of cancer cells 19. A cellular uptake inhibition test was conducted to investigate whether the cellular internalization of CA800-PR in MCF-7 cells occurred via the OATPs-mediated uptake. To clarify the role of serum proteins, we carried out the inhibition assay in the absence of serum. Under the serum-free condition, the membrane transporter proteins OATPs pre-blocked with bromsulphthalein (BSP), which is widely used as an OATP inhibitor, showed no significant inhibition of CA800-PR uptake (Figure S4C). This indicates that the OATP transporters are not involved in this process. And secondly, Usama *et al.*, demonstrated that the tumor accumulation and persistence of the heptamethine cyanine dyes were mediated by the formation of noncovalent or covalent albumin adducts, which can be trapped in tumors via the albumin-mediated endocytosis 20. Although the exact binding mechanism of CA800-PR in tumors still remains unclear, it is clear that the rigid chloro-cyclohexenyl ring in the heptamethine cyanine chain plays an important role in tumor preferential accumulation.

After confirming the tumor retention time, CA800-PR was repeatedly injected every 2 days into the MCF-7 xenograft mice to observe the tumor growth and evaluate the antitumor efficacy for 7 days. Additionally, MDA-MB-231 xenograft mice were also injected under the same condition to fairly compare the antitumor efficacy of CA800-PR (Figure 2B). Interestingly, CA800-PR exhibited obvious tumor suppression in the MCF-7 xenograft mice for 7 days after injection, whereas no reduction of tumor growth was observed in the MDA-MB-231 xenograft mice under the same conditions, consistent with the MTT assay results. Moreover, another type of zwitterionic heptamethine dye ZW800-Cl was also injected in both MDA-MB-231 and MCF-7 xenograft mice, respectively, to compare the tumor targetability and antitumor efficacy (Figure 2C). As expected, ZW800-Cl showed tumor-specific uptake due to possession of the chloro-cyclohexenyl ring, however, there was no significant difference in antitumor effect between two types of xenograft mice after a total of 4 injections for 7 days. By comparing the tumor volumes of ZW800-Cl and CA800-PR in MDA-MB-231 and MCF-7 xenograft mice, only MCF-7 xenograft tumors treated with CA800-PR considerably decreased during a course of treatment for 7 days, compared with that of other treatment groups (Figure 2D). As a note, CA800-PR was tested in the MCF-7 xenografted tumors twice as large as other xenograft models to harvest the tumor samples for quantitative western blot analysis, because once the MCF-7 xenografted tumors less than 100 mm^3^ in size were treated with CA800-PR, the tumors were mostly disappeared during a course of treatment for 7 days. To verify the apoptotic effect of CA800-PR in the MCF-7 xenografted tumors, the PARP expression levels in the tumors treated with ZW800-Cl and CA800-PR were analyzed by western blotting. As expected, the expression level of PARP-1 was significantly reduced in the MCF-7 tumors treated with CA800-PR, compared with that of ZW800-Cl (Figure 2E). Thus, these results demonstrate that CA800-PR can induce apoptotic cell death specifically in the MCF-7 xenografted tumors.

### Molecular mechanism of apoptosis induced by CA800-PR in MCF-7 cells

**Figure 3:**
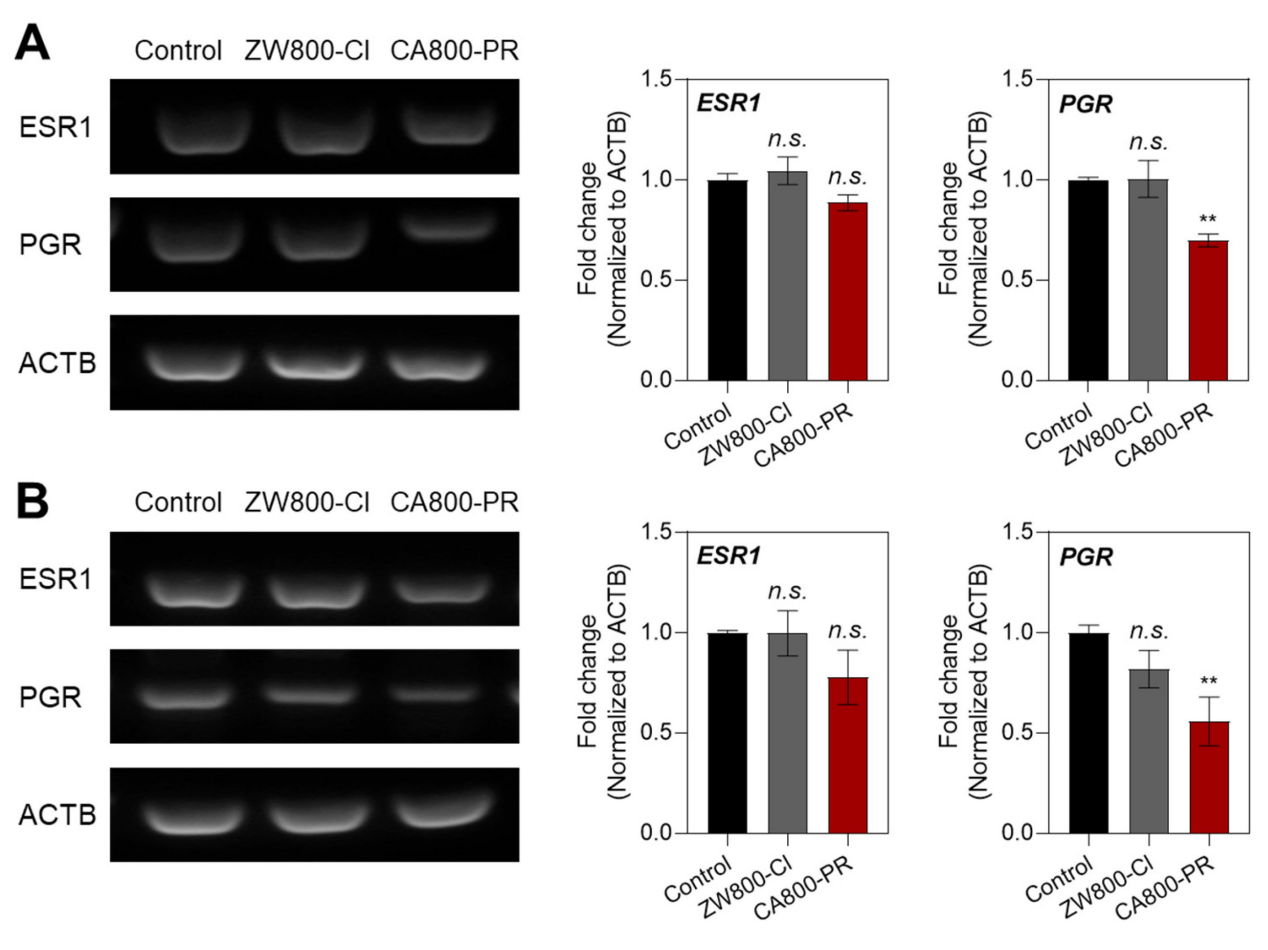
Effect of CA800-PR on mRNA expression in MCF-7 cells and xenograft tumors. A) Semi-quantitative PCR analysis of ESR1, PGR, and ACTB expression in MCF-7 cells. The cells were treated with 100 μM of ZW800-Cl or CA800-PR for 24 h, respectively. B) Semi-quantitative PCR analysis of ESR1, PGR, and ACTB expression in MCF-7 xenograft tumors. Tumor-bearing mice were treated with ZW800-Cl (1.6 mg kg^-1^, *n* = 6) or CA800-PR (1.5 mg kg^-1^, *n* = 6) every 2 days (a total of 4 times in 7 days), respectively. Relative mRNA expression was normalized to ACTB expression. Data are expressed as mean ± S.D. (^**^p < 0.01, *n* = 6).

To further investigate the mechanism of MCF-7-specific apoptosis induced by CA800-PR, we examined the mRNA expression levels of ESR and PGR using a semi-quantitative polymerase chain reaction (PCR) assay, because MCF-7 cells are known to be ESR^+^/PGR^+^ in HR^+^ breast cancers. It is also known that the ESR and PGR are primarily located intracellularly (cytoplasm and nucleus), not on the cell surface, and related to the cell growth and apoptosis depending on their expression levels. After treating with ZW800-Cl and CA800-PR in the MCF-7 cells for 24 h, we confirmed that the mRNA expression levels between ESR1 and PGR showed significant differences in the MCF-7 cells treated with CA800-PR, compared with that of ZW800-Cl (Figure 3A). To reconfirm whether CA800-PR could induce the suppression of PGR rather than ESR in the MCF-7 cells, the mRNA expression levels between ESR1 and PGR were once again determined using the MCF-7 xenografted tumors treated with ZW800-Cl or CA800-PR, respectively. Importantly, the PGR expression was also reduced considerably in the tumor tissues harvested from the CA800-PR-treated mice, while the tumors injected with ZW800-Cl exhibited no significant changes between ESR1 and PGR (Figure 3B). These results suggest that CA800-PR can effectively suppress the PGR activity, regardless of the ESR activity, in the HR^+^ breast cancer.

**Figure 4:**
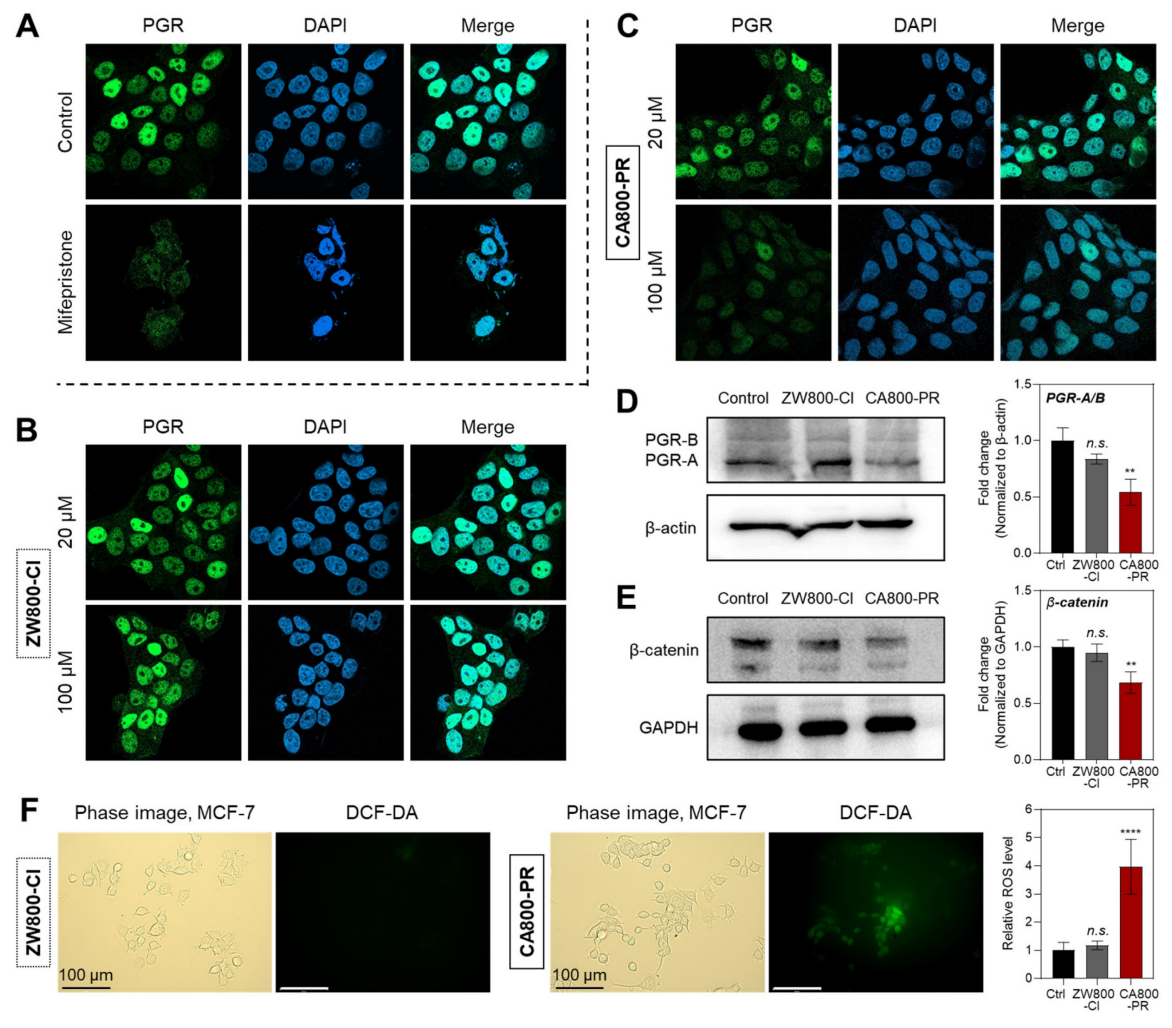
Quantitative analysis of PGR expression in MCF-7 cells. Representative confocal microscopy images showing endogenous PGR expression detected by immunofluorescence staining of MCF-7 cells treated with A) 20 μM of mifepristone, B) 20 or 100 μM of ZW800-Cl, and C) 20 or 100 μM of CA800-PR for 24 h, respectively. Cell nuclei were stained by DAPI (blue). Western blot and quantitative analysis of D) PGR and E) *β*-catenin expression. *β*-actin and GAPDH were taken as the loading control. F) Determination and quantification of cellular ROS by DCF-DA assay. MCF-7 cells were incubated with 20 μM of ZW800-Cl or CA800-PR for 24 h. Scale bars = 100 μm. All fluorescence images have identical exposure times and normalization. MCF-7 cells were treated with 100 μM of ZW800-Cl or CA800-PR for 24 h, respectively. Data are expressed as mean ± S.D. (^**^p < 0.01, ^****^p < 0.0001, *n* = 3).

After confirming the PGR-specific inhibition of CA800-PR, we double-checked the protein expression level of PGR in MCF-7 and T47D cells, as well as noncancerous MCF-10A cells, by immunocytochemistry after treatments with ZW800-Cl or CA800-PR. As shown in Figure 4A, high fluorescence signals of PGR in the nucleus were clearly observed in the control group, because it is known that the PGRs translocate into the nucleus after binding to progesterone. Mifepristone, a representative PGR antagonist, was tested as a standard to compare the expression levels of PGR in each group. As expected, the MCF-7 cells treated with 20 and 100 μM of ZW800-Cl displayed high fluorescence signals in both conditions exactly similar to the control group, consistent with the semi-quantitative PCR results (Figure 4B). Interestingly, the fluorescence intensity of PGR treated with 20 μM of CA800-PR decreased considerably compared with that of the control and ZW800-Cl groups. Eventually, the fluorescence intensity of PGR was more diminished after treatment with 100 μM of CA800-PR, comparable to that of mifepristone (Figure 4C). Additionally, the PGR expression in T47D cells was consistent with that of the MCF-7 cells, while the PGR-negative MCF-10A showed no significant difference in all groups (Figure S7). This result demonstrates that CA800-PR can act as a PGR inhibitor like mifepristone associated with anti-progestational effects.

Since PGR subtype A (PGR-A) is a repressive isoform that can inhibit the transcriptional activity of PGR subtype B (PGR-B), the balance between these two isoforms is crucial for breast cancer cell migration, survival, and response to endocrine therapies 21. Particularly, the imbalance between the PGR-A and PGR-B isoforms, often favoring PGR-A with a relative increase in PGR-A levels, can affect tumor progression and resistance to treatments like tamoxifen 22. In this regard, the expression levels of PGR-A and PGR-B in MCF-7 cells were also identified by western blot analysis after treatments with 100 μM of ZW800-Cl or CA800-PR, respectively. Very importantly, the expression levels of PGR-A significantly decreased in the MCF-7 cells treated with CA800-PR for 24 h, compared with that of ZW800-Cl (Figure 4D). Typically, it is reported that a reduction or loss of PGR expression after chemotherapy is associated with improved disease-free and overall survival in some breast cancer patients, especially those with luminal B-type breast cancer 23. Thus, this result demonstrates that CA800-PR may act as a potent antiproliferative agent in PGR-positive breast cancers. Furthermore, the expression level of *β*-catenin in MCF-7 cells was confirmed by western blot analysis after treatments with 100 μM of ZW800-Cl or CA800-PR, respectively, because it is reported that there is a strong link between breast cancer, PGR signaling, and the canonical Wnt pathway 24. Also, the *β*-catenin activity is commonly upregulated in over 50% of breast cancers and serves as a strong indicator of poor prognosis. As expected, the expression level of *β*-catenin was reduced in the MCF-7 cells treated with CA800-PR for 24 h, compared with that of ZW800-Cl (Figure 4E). This result is correlated with the reduction of PGR expression, because CA800-PR can suppress the PGR expression involved in the Wnt signaling pathway and exert antitumor effects on the MCF-7 cells. Moreover, ROS generation in the MCF-7 cells exposed to stressful conditions after treatments with ZW800-Cl or CA800-PR was examined using the nonfluorescent 2',7'-dichlorodihydrofluorescein diacetate (DCF-DA) method. The CA800-PR induced a significant increase of ROS, which was observed by the oxidized form of DCF emitting green fluorescence, whereas the MCF-7 cells treated with ZW800-Cl showed undetectable fluorescence signals similar to that of the control group (Figure 4F). As an important cellular response to stress conditions, we confirmed the CHOP expression in the MCF-7 cells after treatments with ZW800-Cl or CA800-PR, because CHOP expression can be induced by not only ER stress but also other cellular stresses, such as DNA damage and oxidative stress, resulting in the promotion of apoptosis. The expression level of CHOP protein was significantly upregulated in the MCF-7 cells treated with CA800-PR, compared with that of ZW800-Cl, consistent with the ROS assay (Figure S8).

### Immunogenic cell death and antitumor immune response induced by CA800-PR

**Figure 5:**
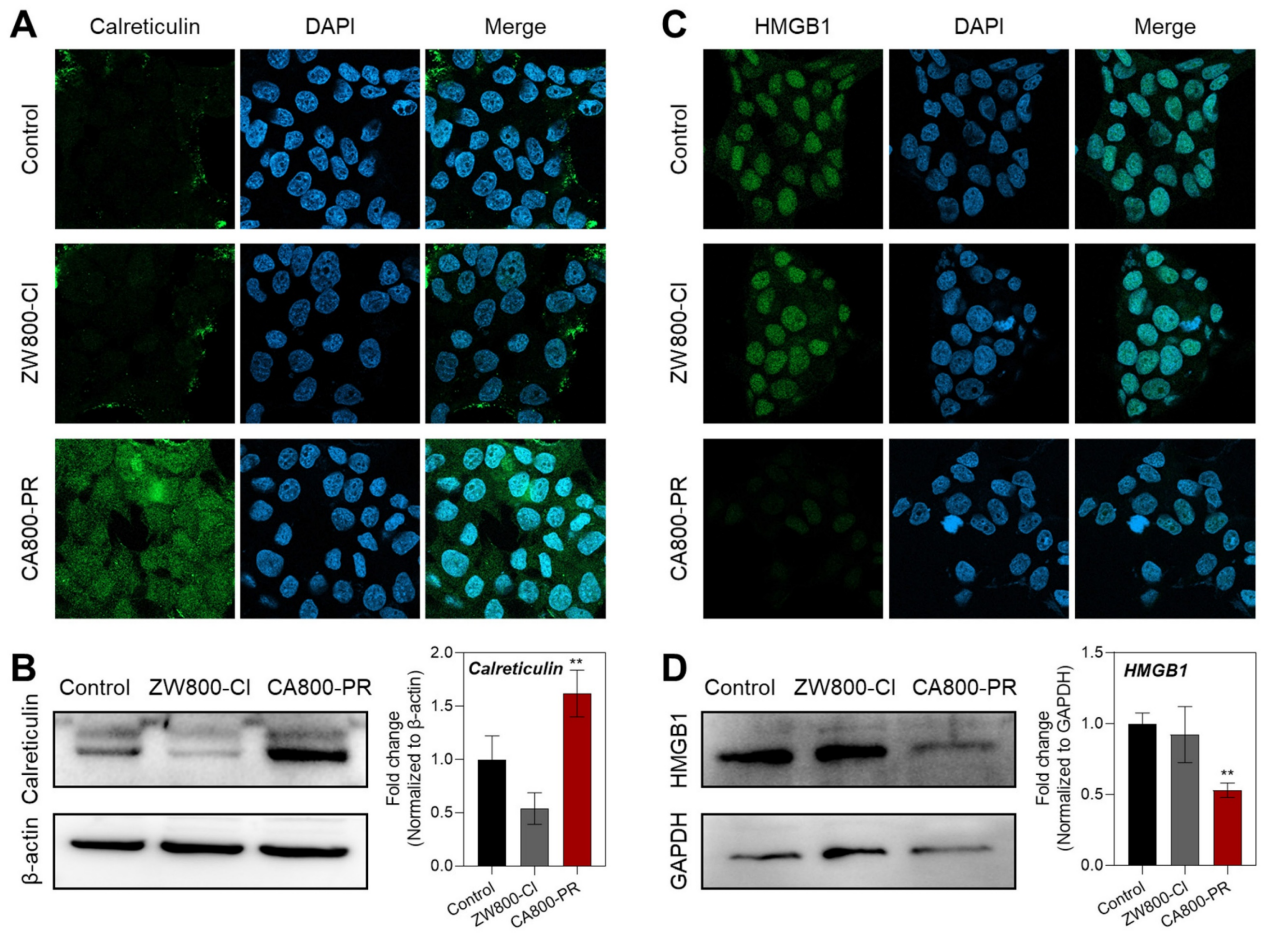
Quantitative analysis of prototypical DAMPs after treatment with CA800-PR in MCF-7 cells. Representative confocal microscopy images showing A) calreticulin and C) HMGB1 expression detected by immunofluorescence staining of MCF-7 cells treated with 100 μM of ZW800-Cl and CA800-PR for 24 h, respectively. Cell nuclei were stained by DAPI (blue). All fluorescence images had identical exposure times and normalization. Western blot and quantitative analysis of B) calreticulin and D) HMGB1 expression. MCF-7 cells were treated with 100 μM of ZW800-Cl or CA800-PR for 24 h, respectively. *β*-actin and GAPDH were taken as the loading control. Data are expressed as mean ± S.D. (^**^p < 0.01, *n* = 3).

As the CA800-PR induced effective apoptotic cell death in the MCF-7 cells, we examined whether the zwitterionic heptamethine cyanine dye CA800-PR could evoke immune responses that would promote antitumor immunity. Through ICD, dying cells can release damage-associated molecular patterns (DAMPs) to attract and activate dendritic cells to initiate ICD. It is well-known that calreticulin, one of the DAMPs, is translocated from the ER to the cell surface owing to ROS-mediated ER stress and act as an “eat-me” signal to promote their phagocytic uptake by the immune system 25. As shown in Figure 5A, an increase in the expression of calreticulin in the cytoplasm was clearly observed in the MCF-7 cells after treatment of CA800-PR for 24 h using immunofluorescence staining and confocal microscopy, compared with that of ZW800-Cl. Additionally, the high expression level of calreticulin after treatment of CA800-PR was reconfirmed by western blotting (Figure 5B). The release of high-mobility group box 1 (HMGB1) protein, another type of DAMPs, was also identified by both immunofluorescence staining and western blotting after treatments of ZW800-Cl or CA800-PR under the same conditions (Figure 5C,D). These results are clear that the MCF-7 cells treated with CA800-PR exhibited very faint green fluorescence and low expression level of HMGB1, compared with that of ZW800-Cl, indicating efficient extracellular release of HMGB1 and confirming strong ICD response in the MCF-7 cells.

**Figure 6:**
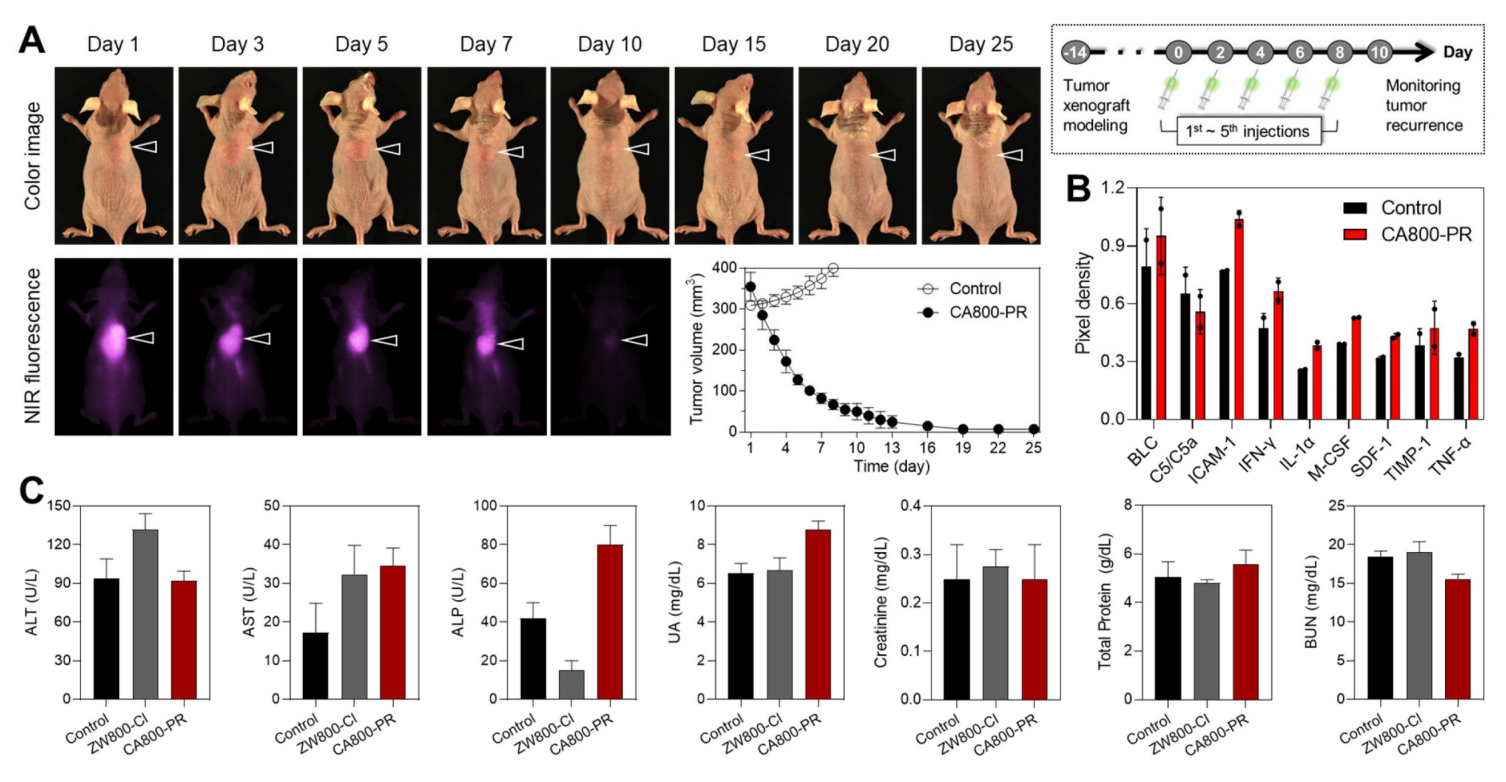
Antitumor efficacy of CA800-PR. A) Antitumor effect of CA800-PR in the MCF-7 xenograft mouse model. Tumor-bearing mice were intravenously injected with CA800-PR (1.5 mg kg^-1^, *n* = 6) every 2 days (5 times in total). The tumor site is indicated by an arrowhead. All NIR fluorescence images have identical exposure times and normalization. Tumor growth rates of each injection group were monitored for 25 days (*n* = 6). B) Mouse cytokine array analysis of multiple cytokines after treatment with CA800-PR. Each cytokine was detected in duplicate. The mouse serum samples were collected at day 10 during treatment with CA800-PR. Pixel density indicates the levels of cytokines in serum samples analyzed using ImageJ software. C) Blood biochemical analysis of mice treated with CA800-PR. The blood samples were collected at day 10 after treatment with CA800-PR.

Motivated by the certain therapeutic efficacy of CA800-PR in the MCF-7 xenograft mice for 7 days, we further monitored tumor growth for 25 days after injection of CA800-PR (1.5 mg kg^-1^, *n* = 6) every 2 days (5 times in total). As a note, the antitumor efficacy of CA800-PR was specially tested in the MCF-7 xenografted tumors greater than 300 mm^3^ in size, because the growth rate of tumors reached 200 mm^3^ in size markedly decreased during a course of treatment for 7 days, as shown in Figure 2B,D. Remarkably, Figure 6A shows that intravenously injected CA800-PR rapidly and effectively suppressed tumor growth within 10 days, compared with that of the control group. Subsequently, tumor regrowth was not observed until the next 15 days, suggesting the broader therapeutic applicability of CA800-PR. To better understand the antitumor effect of CA800-PR in the MCF-7 xenograft mice, we examined cytokine expression by collecting the serum samples at day 10 during treatment with CA800-PR (Figure S9). Since cytokines like IFN-γ and TNF-α play an important role in creating an immunogenic tumor microenvironment (TME), the cytokine array results revealed that there was an increase in the expression of pro-inflammatory cytokines such as IFN-γ, IL-1α, and TNF-α (Figure 6B). Meanwhile, serum biochemical parameters, including alanine aminotransferase, aspartate aminotransferase, alkaline phosphatase, uric acid, creatinine, total protein, and blood urea nitrogen were analyzed to assess systemic toxicity of CA800-PR during a course of treatment for 10 days. As expected, no significant differences in the treatment group of CA800-PR were detected as compared to mice in the control group (Figure 6C).

**Figure 7:**
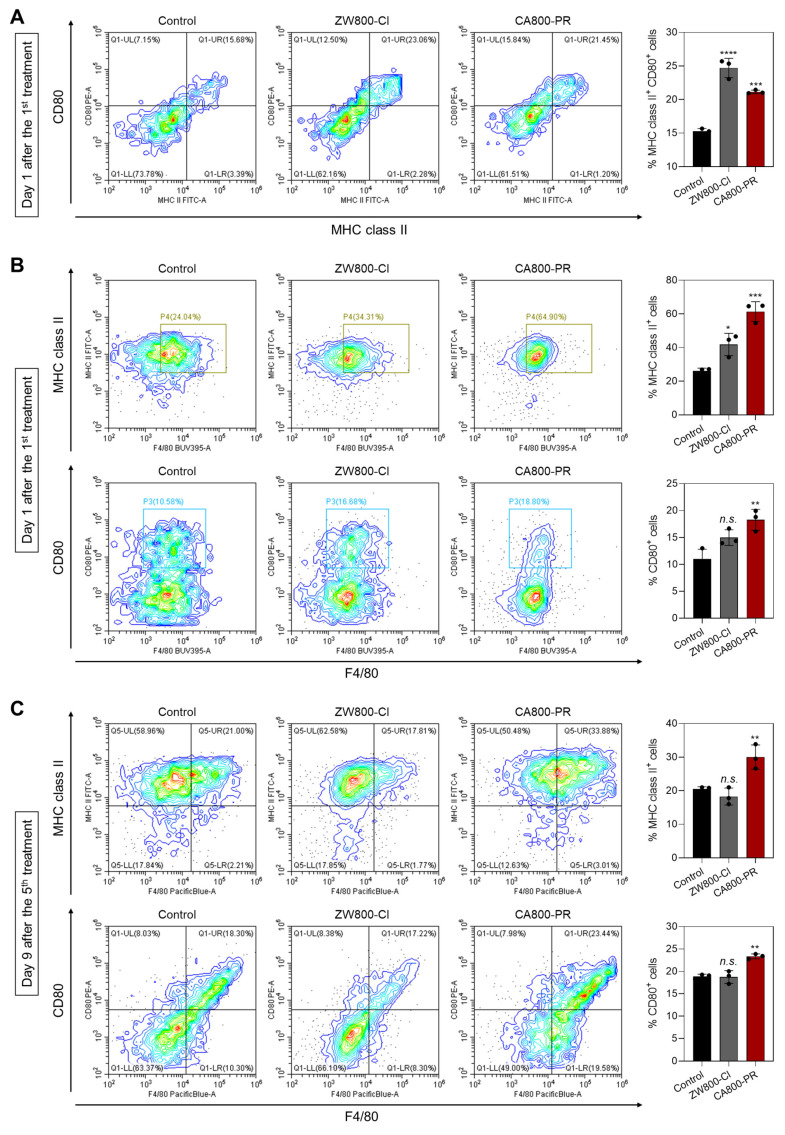

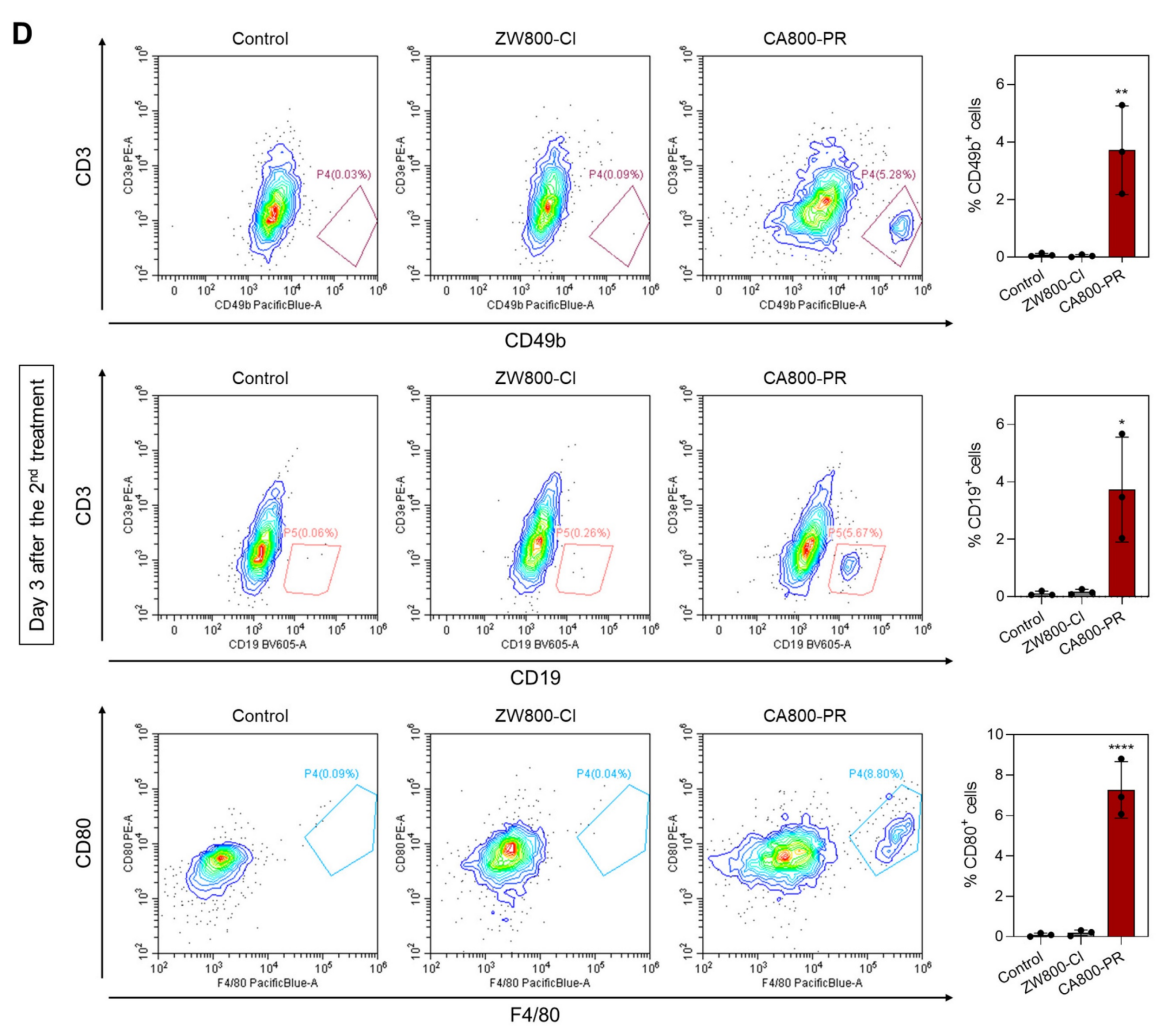
Identification of immune subsets after treatments with ZW800-Cl and CA800-PR. A) Flow cytometry assay and quantification of matured DCs (MHC class II^+^CD80^+^) in BMDCs (*n* = 3) measured at day 1 after the 1^st^ treatments with ZW800-Cl or CA800-PR, respectively. B, C) Flow cytometry assay and quantification of M1 macrophages (F4/80^+^MHC class II^+^CD80^+^) in the spleen measured at day 1 and 9 after the 1^st^ and 5^th^ treatments with ZW800-Cl or CA800-PR, respectively. D) Flow cytometry assay and quantification of NK cells, B cells, and M1 macrophages (CD49b^+^CD19^+^CD80^+^) in the tumor measured at day 3 after the 2^nd^ treatment with ZW800-Cl or CA800-PR, respectively. Data are expressed as mean ± S.D. (^*^p < 0.05, ^**^p < 0.01, ^***^p < 0.001, ^****^p < 0.0001, *n* = 3).

Finally, we further investigated the immune response induced by the treatment of CA800-PR. It is well-known that dendritic cells (DCs) play an irreplaceable role in the adaptive immune process. ICD induces the accumulation and maturation of DCs, which further stimulates T-cell maturation and initiates the immune cycle to achieve tumor killing. The cell surface of mature DCs expresses MHC class II and co-stimulatory molecules (CD80/CD86), which are required to activate the T-cell response 26. Hence, we examined the proportion of mature DCs in the bone marrow derived dendritic cells (BMDCs) by flow cytometry after treatments with ZW800-Cl or CA800-PR for 24 h (Figures 7A and S10). The results showed that the proportions of mature DCs in the groups treated with ZW800-Cl and CA800-PR were 1.47-fold and 1.37-fold higher than that of the control group. Since it is known that M1 macrophages also express high levels of surface markers, such as CD80 and MHC class II, which are associated with antigen presentation and T-cell activation, we also examined M1 marker protein expression in the spleen harvested at days 1 and 9 after treatments with ZW800-Cl or CA800-PR, respectively (Figures 7B,C and S11). As expected, the expression levels of M1 marker proteins, MHC class II and CD80, in the spleen were significantly increased at both day 1 and 9 when compared to those in the control and ZW800-Cl groups. Interestingly, the DC maturation could be induced in both conditions treating ZW800-Cl or CA800-PR, however, the M1 macrophages were activated only by the treatment of CA800-PR. Furthermore, we confirmed the expression of NK cells, B cells, and M1 macrophages in the tumors collected at day 3 after treatments with ZW800-Cl or CA800-PR, respectively (Figures 7D and S12). As strong evidence of immune cell infiltration, the expression levels of CD49b, CD19, and CD80 proteins in the tumor were significantly increased only in the treatment group of CA800-PR, compared with that of other groups. These results demonstrate that the synergistic effects of PGR inhibition and ICD effectively induced DC maturation and upregulated M1 macrophage percentage, with the potential to reverse the immunosuppressive TME and enhance the tumor suppression. Taken together, the treatment of CA800-PR can promote antitumor immunity by inducing ICD associated with the release of pro-inflammatory cytokines, thereby achieving safe and effective cancer immunotherapy.

## Conclusion

In summary, we have synthesized a newly designed heptamethine cyanine dye CA800-PR to use in a simple and more effective treatment of HR^+^ breast cancers by inhibiting PGR and inducing ICD. Based on the “structure-inherent cancer targeting” strategy, the water-soluble zwitterionic small molecule dye CA800-PR successfully showed tumor-specific targeting, NIR fluorescence imaging, and tumor-targeted therapy in an MCF-7 xenograft mouse model. This multifunctional theranostic agent CA800-PR could suppress the PGR activity and produce intracellular ROS through Golgi fragmentation. Consequently, the intracellular stress could lead to release DAMPs into the extracellular space, which activate dendritic cells and stimulate antitumor immunity. As a result of the activation of an immunomodulatory response, we confirmed a significant increase in the presence of antitumor/pro-inflammatory MHC class II^+^ CD80^+^ M1-type macrophages during the course of treatment. To date, most other studies have been focused mainly on targeting ESR signaling for the typical treatment of HR^+^ breast cancers. Herein, we provide not only a simple and effective strategy to develop multifunctional small molecule theranostic agents but also an understanding of the PGR inhibition mechanism induced by Golgi fragmentation in MCF-7 cancer cells and xenograft tumors. For further study, the *in vivo* therapeutic efficacy of CA800-PR in other types of HR^+^ breast cancers, including ESR^-^/PGR^+^, ESR^+^/PGR^-^, PGR^+^/HER2^+^, and PGR^+^/HER2^-^ breast cancers, is now under investigation.

## Materials and Methods

### Chemicals and synthesis

All chemicals and solvents were of American Chemical Society grade or high-performance liquid chromatography (HPLC) purity. Starting materials were purchased from Sigma-Aldrich (St. Louis, MO, USA) and used as received without further purification. Final products were separated by preparative HPLC system equipped with a PrepLC 150 mL fluid handling unit, a manual injector (Rheodyne 7725i) and a 2487 dual wavelength absorbance detector (Waters, Milford, MA, USA). ^1^H NMR spectrum was recorded on a Bruker Avance (400 MHz) spectrometer. The accurate mass of the purified product was analyzed by the Dionex UltiMate^TM^ 3000 mass spectrometry system (Thermo Scientific, Waltham, MA, USA). The water-soluble zwitterionic heptamethine cyanine dyes, ZW800-Cl and CA800-PR, were synthesized as follows:

*2-[(E)-2-[(3E)-2-chloro-3-{2-[(2E)-3,3-dimethyl-5-sulfonato-1-[3-(trimethylazaniumyl)propyl]-2,3-dihydro-1H-indol-2-ylidene]ethylidene}cyclohex-1-en-1-yl]ethenyl]-3,3-dimethyl-1-[3-(trimethylazaniumyl)propyl]-3H-indol-1-ium-5-sulfonate bromide (**ZW800-Cl**)*. A mixture of 4-hydrazinobenzene sulfonic acid **1** (6 g, 31.9 mmol), and 3-methyl-2-butanone **3** (4.6 mL, 41.8 mmol) in glacial acetic acid (50 mL) was heated at 118 °C under a nitrogen atmosphere in a round flask for 18 h. The crude product was filtered and collected as a pink solid after precipitation in ethyl acetate (6.5 g, 80%). 2,3,3-trimethylindolenine-5-sulfonic acid **4** (0.2 g, 0.83 mmol) and (3-bromopropyl)trimethyl-ammonium bromide **6** (0.25 g, 0.96 mmol) in toluene (5 mL) were heated at 70 °C for 48 h under a nitrogen atmosphere. The mixture was cooled to ambient temperature and the solvent was decanted. The crude mixture was recrystallized in acetonitrile. The collected solid was used directly in the next step without further purification (0.16 g, 46%). A mixture of heterocyclic salt **7** (0.15 g, 0.36 mmol), Vilsmeier-Haack reagent **9** (0.05 g, 0.14 mmol), and anhydrous sodium acetate (0.04 g, 0.48 mmol) in absolute ethanol (5 mL) was heated under reflux for 6 h. The reaction mixture was cooled to room temperature and then filtered, washed with ethanol and methanol, and collected as a green solid **10** (ZW800-Cl; 0.1 g, 88%). Accurate mass HRMS (ESI) m/z [M]^+^ calculated for [C_42_H_58_ClN_4_O_6_S_2_]^+^ 813.3480, found [M+H]^+^ 815.0366.

*5-carboxy-2-[(E)-2-[(3E)-3-{2-[(2E)-5-carboxy-3,3-dimethyl-1-[3-(trimethylazaniumyl) propyl]-2,3-dihydro-1H-indol-2-ylidene]ethylidene}-2-chlorocyclohex-1-en-1-yl]ethenyl]-3,3-dimethyl-1-[3-(trimethylazaniumyl)propyl]-3H-indol-1-ium bromide (**CA800-PR**)*. Glacial acetic acid (15 mL) was added to a mixture of 4-hydrazinobenzoic acid **2** (1 g, 6.6 mmol) and 3-Methyl-2-butanone **3** (1.1 mL, 9.9 mmol) in a round-bottomed flask fitted with a condenser. The brown suspension was refluxed for 8 h, and the solvent was removed under reduced pressure with a rotavapor. The residue was redissolved into a clear solution using water and methanol (10 mL, 90/10 v/v%). Undissolved material was filtered off, the filtrate was allowed to stand at room temperature, and the yellow crystal **5** (0.9 g, 67%) was collected by filtration. (3-bromopropyl)trimethyl-ammonium bromide **6** (0.91 g, 2.17 mmol) followed by toluene (10 mL) were added to a dried flask containing carboxylated indole **5** (0.5 g, 2.5 mmol), and the suspension was refluxed for 18 h. The precipitate intermediate **8** was collected by filtration and washed with acetonitrile and ethyl acetate. The collected solid was used directly in the next step without further purification (0.6 g, 70%). A mixture of heterocyclic salt **8** (0.11 g, 0.29 mmol) and Vilsmeier-Haack reagent **9** (0.046 g, 0.13 mmol), and anhydrous sodium acetate (0.04 g, 0.48 mmol) in absolute ethanol (5 mL) was heated under reflux for 6 h, respectively. The reaction mixtures were cooled to ambient temperature, and then dried by rotovap to remove the reaction solvent, and purified by preparative HPLC system (Waters) and obtained as a dark-green solid **11** (CA800-PR; 0.08 g, 75%). Accurate mass HRMS (ESI) m/z [M]^+^ calculated for [C_44_H_60_ClN_4_O_4_]^+^ 743.4286, found [M+H]^+^ 745.1432.

### Optical and physicochemical property analyses

All optical measurements were performed in PBS (pH 7.4). The absorption spectra of ZW800-Cl and CA800-PR were recorded using a fiber optic FLAME absorbance and fluorescence (200-1025 nm) spectrometer (Ocean Optics, Dunedin, FL, USA). The molar extinction coefficient was calculated using the Beer-Lambert equation. To determine the fluorescence quantum yield (*Φ*), indocyanine green (ICG) dissolved in dimethyl sulfoxide (DMSO, *Φ* = 13%) 27 was used as a calibration standard under the condition of matched absorbance at 770 nm. The fluorescence emission spectra of ZW800-Cl and CA800-PR were analyzed using a SPARK^®^ 10M microplate reader (Tecan, Männedorf, Switzerland). *In silico* calculations of the distribution coefficient (log*D* at pH 7.4) and the topological polar surface area (TPSA) were performed using Marvin and JChem calculator plugins (ChemAxon, Budapest, Hungary).

### Cytotoxicity assay

The human colorectal adenocarcinoma cell line (HT-29, Cat# HTB-38, RRID:CVCL_0320), the human large-cell lung carcinoma cell line (NCI-H460, Cat# HTB-177, RRID:CVCL_0459), the human breast cancer cell lines (MDA-MB-231, Cat# HTB-26, RRID:CVCL_0062; MCF-7, Cat# HTB-22, RRID:CVCL_0031; T47D, Cat# HTB-133, RRID:CVCL_0553), the mouse embryonic fibroblast cell line (NIH/3T3, Cat# CRL-1658, RRID:CVCL_0594), and the human normal breast epithelial cell line (MCF-10A, Cat# CRL-10317, RRID:CVCL_0598) were obtained from the American Type Culture Collection (ATCC, Manassas, VA, USA). Cells were maintained in Roswell Park Memorial Institute (RPMI) 1640 media containing fetal bovine serum (FBS, pH 7.4), penicillin, streptomycin, and amphotericin B (Welgene, Daegu, South Korea) on a culture plate. The cultured cells were stored in a humidified incubator set to 5% CO_2_ at 37 °C. When the cells reached a confluence of approximately 80%, cell toxicity and proliferation were evaluated using the 3-(4,5-dimethylthiazol-2-yl)-2,5-diphenyltetrazolium bromide (MTT) assay. Cells were seeded onto 96-well plates (5 × 10^3^ cells per well). To evaluate the cytotoxicity depending on the concentrations of each sample, the cells were treated with CA800-PR (2, 10, 20, 50, and 100 μM) for 24 h. After treatment, the incubation cell medium was replaced with 100 μL of fresh medium, and 10 μL of the MTT solution was directly added to each 100 μL well. Subsequently, the plates were then incubated for 4 h at 37 °C in a humidified 5% CO_2_ incubator. After adding DMSO, the plates were placed in a microplate reader (SPARK^®^ 10M, Tecan) to measure the absorption intensity at 570 nm. Cell viability was calculated using the following formula: cell viability (%) = (*A*_sample_ - *A*_blank_)/(*A*_control_ - *A*_blank_) × 100, where *A* is the average absorbance.

### Live cell imaging and subcellular localization of CA800-PR

The cultured cells were treated with 100 μM of CA800-PR and incubated for 24 h at 37 °C in a humidified 5% CO_2_ incubator. After the treatment, nucleus, Mito, ER, and Golgi apparatus were stained with DAPI (Cat# D9542, Sigma-Aldrich), Mito-Tracker Green (Cat# M7514, Thermo Fisher Scientific), ER-Tracker Green (Cat# E34251, Thermo Fisher Scientific), Lyso-Tracker Green (Cat# L7526, Thermo Fisher Scientific), and Golgi-Tracker Green (Cat# B8813, APExBIO Technology, Houston, TX, USA) for 5-30 min according to the manufacturer's instructions. The cells were then washed with DPBS and imaged using the fluorescence microscope (Nikon). All fluorescence images had identical exposure time and normalization.

### Cellular uptake inhibition assay

To confirm whether the cellular internalization of CA800-PR in MCF-7 cells occurred via OATPs-mediated uptake under the absence of serum, MCF-7 cells were cultured with serum-free medium and pretreated with 250 μM of BSP (OATP inhibitor; 167207, Sigma-Aldrich) for 30 min. Cells were then incubated with 100 μM of CA800-PR at 37 °C for 1 h. After washing, the cells were imaged using the fluorescence microscope (Nikon).

### Intracellular ROS detection assay

MCF-7 cells were incubated with 20 μM of ZW800-Cl and CA800-PR for 24 h at 37 °C in a humidified 5% CO_2_ incubator. Subsequently, the cells were washed with PBS and treated with a 100 μM of DCF-DA (Thermo Fisher Scientific) for 30 min at 37 °C. Cellular ROS production was commonly estimated by nonfluorescent DCF-DA, which permeates the cell membrane and reacts with reactive oxygen to give a DCF form emitting green fluorescence. After the treatment of DCF-DA, the cells were repeatedly washed with PBS and then observed under the fluorescence microscope (Nikon).

### Quantitative PCR

Total RNA was isolated using the TRIzol Reagent (Cat# T9424, Sigma-Aldrich) according to the manufacturer's instructions, and 1 μg of RNA was reverse transcribed using random hexamer (Cat# SO142, Thermo Fisher Scientific) and RevertAid reverse transcriptase (Cat# EP0441, Thermo Fisher Scientific). The complementary DNA (cDNA) was used in semi-quantitative PCR. PCR amplification of the cDNA products was performed with nTaq (Mg2+Plus) (Enzynomics, Daejeon, South Korea) and primer pairs. Amplified products were separated by 2% agarose gel electrophoresis. mRNA levels were normalized using ACTB as an internal control. The primer sequences are as follows: ESR1 (forward: 5′-AGG GTG GCA GAG AAA GAT TG-3′; reverse: 5′-GTG GCT GGA CAC ATA TAG TCG-3′), PGR (forward: 5′-ACA CAA AAC CTG ACA CCT CC-3′; reverse: 5′-CTC CAT CCT AGA CCA AAC ACC-3′), ACTB (forward: 5′- ACC ACA CCT TCT ACA ATG AGC-3′; reverse: 5′-GAT AGC ACA GCC TGG ATA GC-3′). The experiments were performed in triplicate and repeated thrice with consistent results.

### Western blot analysis

The whole cell or tissue extracts were prepared by lysing the treated cells in RIPA buffer (Cat# RC2002-050-00, Biosesang, Yongin, South Korea). Supernatants of cell lysis were collected after centrifugation and mixed with Laemmli sample buffer (Cat# 1610737, Bio-Rad, Hercules, CA, USA), followed by heating to 95 °C for 3 min to make the protein fully denatured. Subsequently, the protein (40 μg) was added into the wells and proteins were separated by the 8-15% sodium dodecyl sulfate polyacrylamide gel electrophoresis (SDS-PAGE). After electrophoresis, the SDS-PAGE gel carrying proteins was then transferred onto a polyvinylidene difluoride membrane (Merck Millipore, Burlington, MA, USA) activated by methanol. The membranes were then blocked with 5% bovine serum albumin (BSA, Cat# A9418, Sigma-Aldrich) for 2 h at room temperature, followed by incubation with specific primary antibodies (1:1000) overnight at 4 °C. Primary antibodies against PARP (Cat# 9542, Cell Signaling, Beverly, MA, USA), CHOP (Cat# 2895, Cell Signaling), Progesterone Receptor A/B (Cat# 8757, Cell Signaling), *β*-catenin (Cat# 9562, Cell Signaling), Calreticulin (Cat# 12238, Cell Signaling), HMGB1 (Cat# 3935, Cell Signaling), and *β*-actin (Cat# sc-47778, Santa Cruz, Dallas, TX, USA) were used. The secondary antibody labeled with horseradish peroxidase was combined with the primary antibody and incubated for 2 h at room temperature. After washing the membrane, the signals were detected using an enhanced chemiluminescent substrate solution (Thermo Fisher Scientific). Finally, bands were captured using a LuminoGraph III Lite (WSE-6370, Atto, Tokyo, Japan) and quantified using ImageJ software. Protein expression was normalized to *β*-actin.

### Immunofluorescence staining and confocal imaging

MCF-7, T47D, and MCF-10A cells were seeded onto coverslips at a density of 1 × 10^5^ cells/well in 12-well plates. 20 or 100 μM of ZW800-Cl and CA800-PR was incubated in MCF-7 cells for 24 h. The cells were washed with PBS before being fixed in 4% paraformaldehyde for 20 min. After fixation, the cells were rinsed with PBS and permeabilized with 0.1% Triton X-100 for 5 min. Subsequently, the cells were incubated in a 5% BSA solution for 1 h to block non-specific binding sites. After washing with PBS, the cells were exposed to primary antibodies for overnight at 4 °C. The cells were rewashed with PBS, followed by treatment with fluorescent-labeled secondary antibodies and nuclear staining using DAPI for 5 min. Once the coverslips were mounted onto the glass slides using a mounting medium, the immunofluorescence and confocal imaging were examined using a confocal fluorescence microscope (ZEISS LSM 900, Carl Zeiss, Germany) at × 63 magnification.

### Xenograft mouse model

Animal experiments were carried out in accordance with the guidelines approved by the Chonnam National University Animal Research Committee (CNU IACUC-H-2024-75). Female athymic nude mice (6 weeks old and ≈25 g) were received from Orient Bio (Gwangju, South Korea) to prepare human tumor xenograft models. The cultured MCF-7 and MDA-MB-231 cancer cells were dispersed in PBS and inoculated subcutaneously into the mouse upper right flank area (1 × 10^6^ cells per mouse). Finally, mice bearing subcutaneous tumors sized with an average diameter of 1 cm were subjected to intravenous injection of ZW800-Cl or CA800-PR, respectively. The tumor bearing mice were anesthetized at specific time points for real-time whole body imaging. *In vivo* NIR fluorescence imaging was performed using a FOBI imaging system (NeoScience, Deajeon, South Korea). The nude mice (6 mice per treatment group) were imaged for 48 h after intravenous injection to confirm the time-dependent accumulation of ZW800-Cl or CA800-EV in tumors. All NIR fluorescence images were normalized identically for all conditions.

### Cytokine array

Mice bearing MCF-7 xenografts were treated with PBS or CA800-PR and evaluated for cytokine release using a Mouse Cytokine Array Kit (Cat# ARY006, R&D Systems, Minneapolis, MN, USA), according to the manufacturer's instructions. Briefly, the total proteins of mouse serum were extracted. The array membranes were incubated with block buffer for 1 h and incubated with serum overnight at 4 °C. The membranes were washed and then incubated with a streptavidin-HRP for 2 h at room temperature. Finally, the images were captured using a LuminoGraph III Lite (WSE 6370, Atto). The intensity score of each array spot was analyzed using ImageJ software.

### BMDC isolation and flow cytometry

BMDCs were isolated from the bone marrow of C57BL/6 mice according to established protocols. Femur bones were isolated from healthy C57BL/6 mice. Then, both ends of the femur were cut, and the bone marrow was flushed out by slowly injecting RPMI 1640 culture medium. The collected bone marrow was filtered through a 70 μm cell strainer to a 50 mL centrifuge tube, followed by centrifugation at 250 × *g* for 8 min and subsequent lysis of red cells. The obtained cells were resuspended in RPMI 1640 culture medium (10 mL) supplemented with 20 ng/mL of granulocyte-macrophage colony-stimulating factor (GM-CSF) and incubated at 37 °C in a humidified 5% CO_2_ incubator. On day 3, an additional fresh culture medium (10 mL) supplemented with 20 ng/mL of GM-CSF was added and incubated for another 3 days. Afterward, BMDCs were harvested by collecting the non-adherent and loosely adherent cells from the suspension. The collected BMDCs were treated with 20 μM of ZW800-Cl or CA800-PR for 24 h. Finally, the population of mature BMDCs was analyzed using a flow cytometer (CytoFLEX, Beckman Coulter, Indianapolis, IN, USA).

### Immune cell isolation and flow cytometry

Mice bearing MCF-7 xenografts were treated with PBS, ZW800-Cl, or CA800-PR for 9 days and subsequently sacrificed to excise spleens or tumors. The collected spleens or tumors were cut into small pieces and ground into tissue suspension in an ice box. The tissue suspension was filtered through 70 μm cell strainers to obtain single-cell suspension, followed by treatment with red blood cell lysis buffer and washing with PBS. The harvested cells were then stained with fluorescence-labeled antibodies according to the manufacturer's instructions. The antibodies were listed as follows: CD49b (Cat# 14-5971-85, Thermo Fisher Scientific), CD19 (Cat# 12-0199-42, Thermo Fisher Scientific), CD80 (Cat# 12-0801-82, Thermo Fisher Scientific), MHC class II (Cat# 11-5321-82, Thermo Fisher Scientific), and F4/80 (Cat# 565411, BD Biosciences, San Jose, CA, USA). All antibodies used in the experiments were diluted in 1:200. The labelled cells were analyzed using a flow cytometer (CytoFLEX).

### Assessment of safety *in vivo*

To evaluate the potential toxicity of CA800-PR *in vivo*, blood samples were collected at day 10 after treatment with CA800-PR. The collected blood samples were centrifuged at 4500 rpm at 4 °C. Subsequently, the supernatant was separated and stored at -80 °C for analysis of blood biochemical parameters.

### Statistical analysis

Statistical analysis was performed using a one-way analysis of variance (ANOVA) followed by Tukey's multiple comparisons test. Differences were considered to be statistically significant at a level of *p* < 0.05. Results were presented as the mean ± S.D. for all the analyses. Statistical analysis and curve fitting were performed using Prism software (GraphPad, San Diego, CA, USA).

## Supplementary Material

Supplementary figures.
