# Supplementary material for: A tumor-targeted heptamethine cyanine dye induces suppression of progesterone receptor activity to treat hormone receptor-positive breast cancer
Source: Theranostics. 2026 Jan 1;16(6):2764–79. doi: 10.7150/thno.126396 (PMC12775649; doi:10.7150/thno.126396)
Supplement: Supplementary file 1 — Supplementary figures. [file thnov16p2764s1.pdf]

A tumor-targeted heptamethine cyanine dye induces suppression of progesterone receptor activity to treat hormone receptor-positive breast cancer

Yoonbin Park[1,2,†], Sang-Hyo Kim[1,2,†], Moon Suk Kim[3,*], Hoon Hyun[1,2,*]

[1] Department of Biomedical Sciences, Chonnam National University Medical School, Hwasun 58128, South Korea
[2] BioMedical Sciences Graduate Program (BMSGP), Chonnam National University, Hwasun 58128, South Korea
[3] Department of Molecular Science and Technology, Ajou University, Suwon 16499, South Korea

[†] Yoonbin Park and Sang-Hyo Kim contributed equally to this work.

[*] Corresponding authors: Moon Suk Kim, E-mail: moonskim@ajou.ac.kr; Hoon Hyun, E-mail: hhyun@jnu.ac.kr

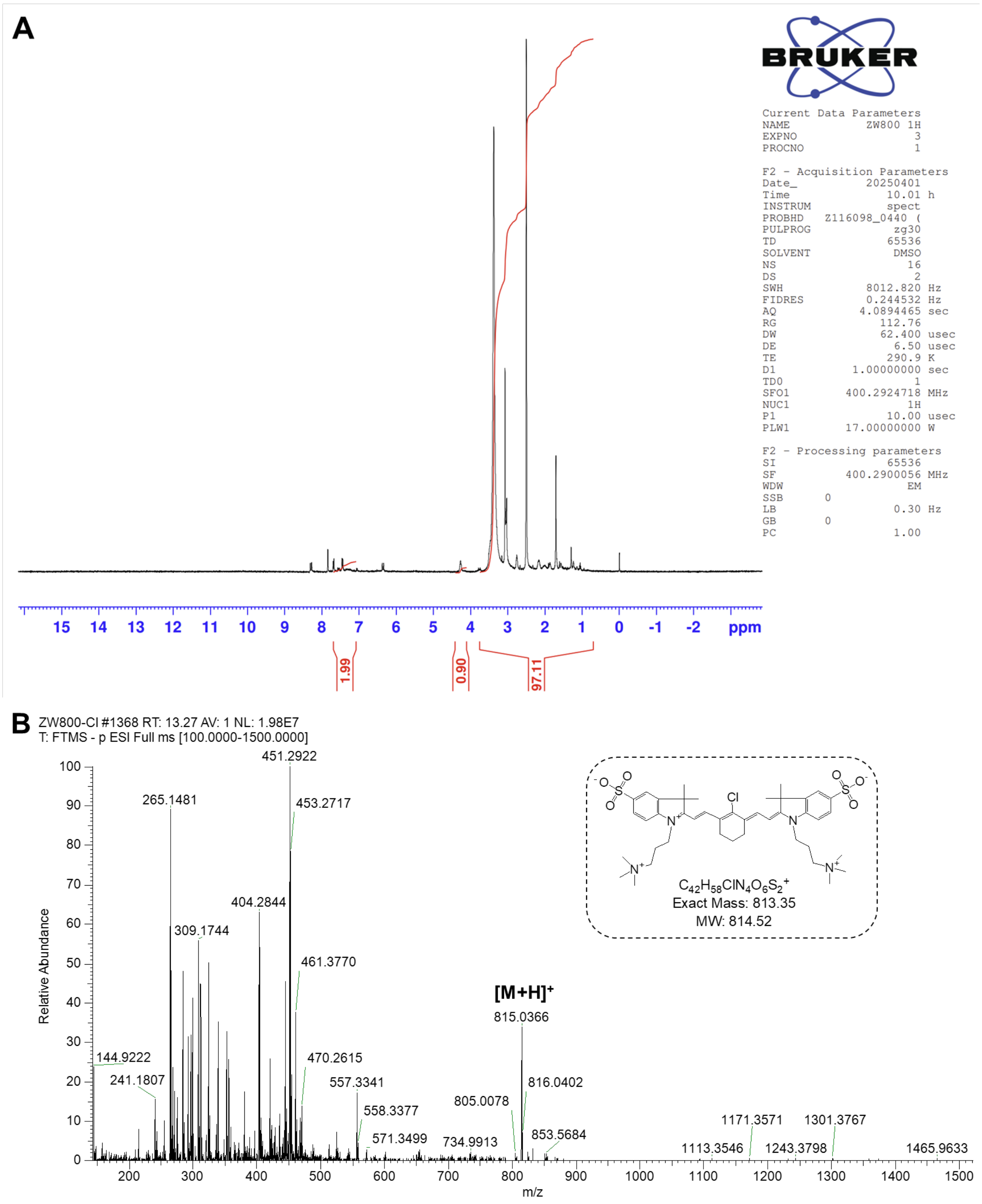

Figure S1. A) ^{1}H NMR and B) mass spectra of ZW800-Cl.

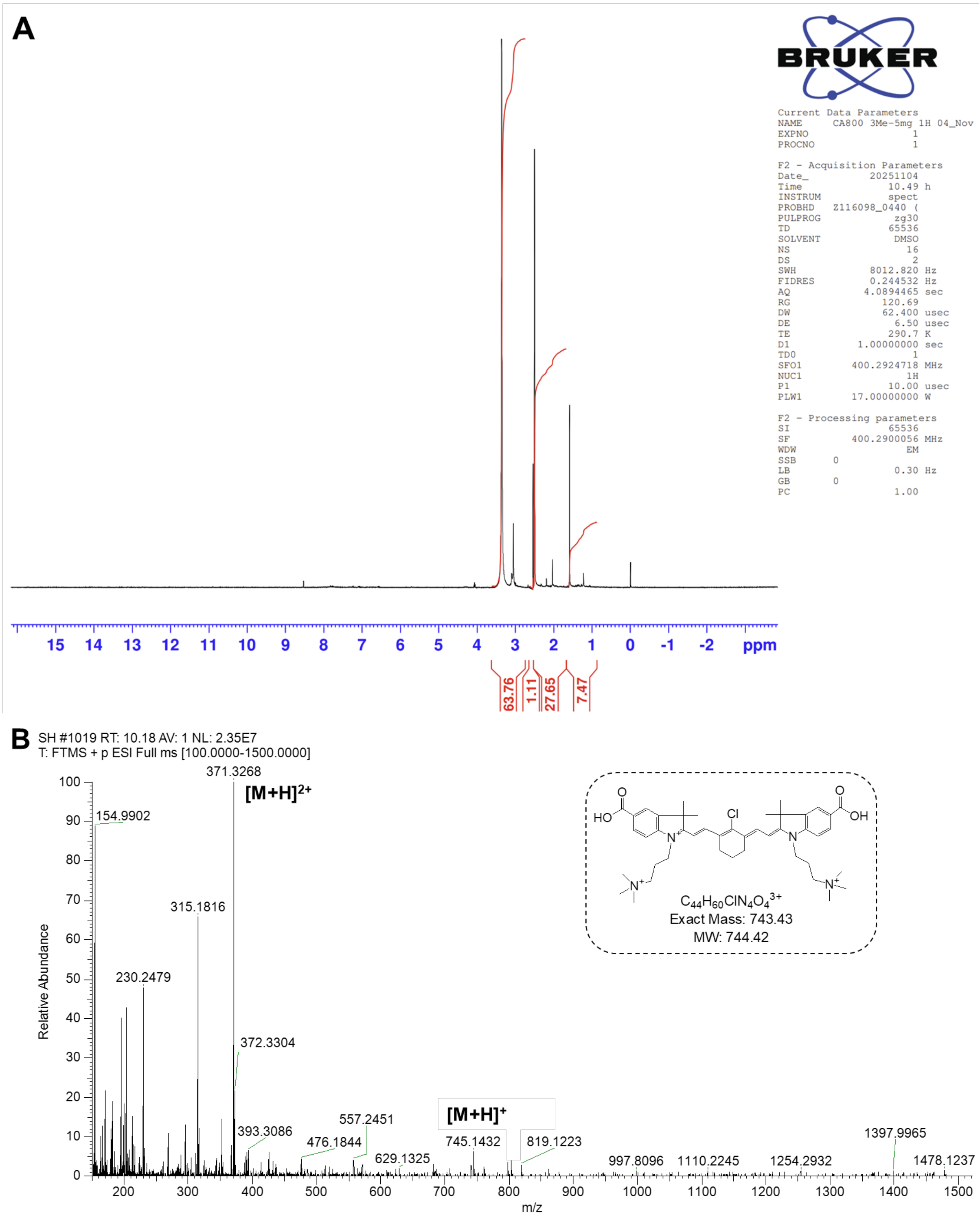

Figure S2. A) ^{1}H NMR and B) mass spectra of CA800-PR.

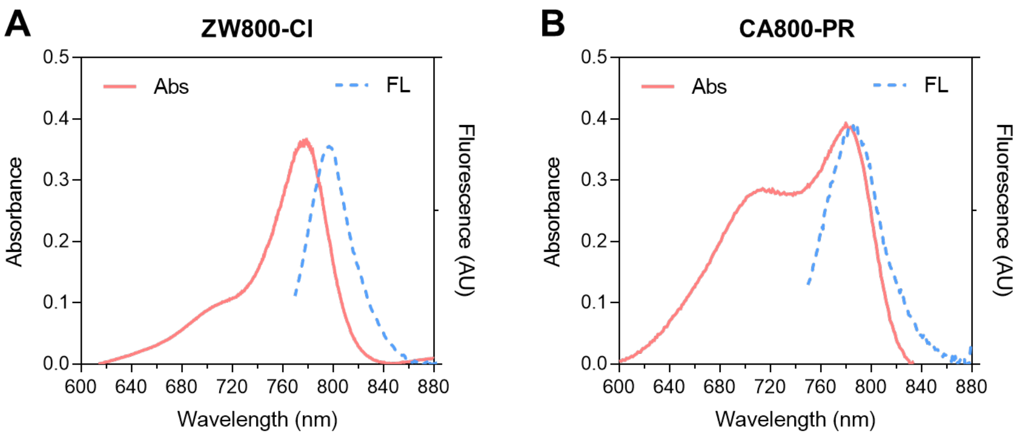

Figure S3. Absorption and fluorescence emission spectra of A) ZW800-Cl and B) CA800-PR measured in PBS at pH 7.4.

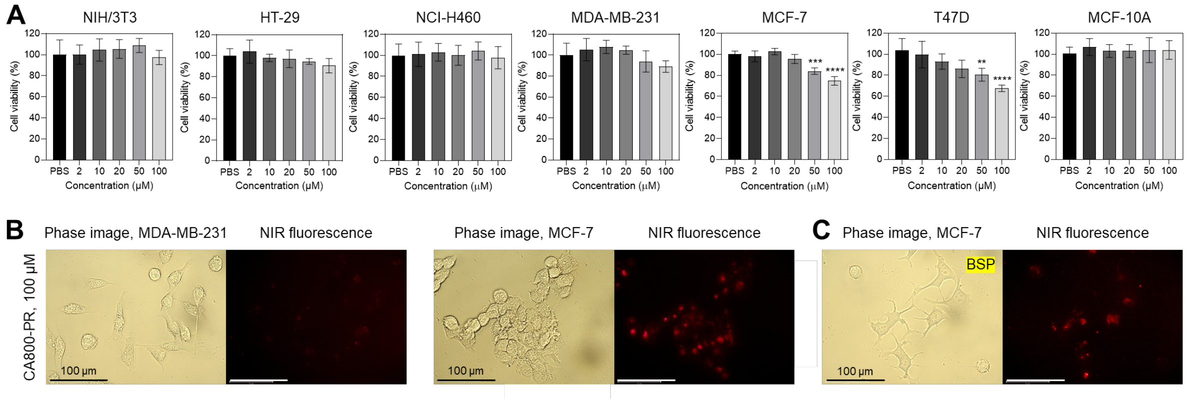

Figure S4. A) Cell viability assay of CA800-PR using NIH/3T3 fibroblasts, HT-29 colon cancer cells, NCI-H460 lung cancer cells, MDA-MB-231, MCF-7 breast cancer cells, T47D breast cancer cells, and MCF-10A normal breast epithelial cells, respectively. Percentage cytotoxicity is determined after 24 h of treatment with various concentrations of CA800-PR. Data are expressed as mean ± S.D. ($^{**}p < 0.01$, $^{***}p < 0.001$, $^{****}p < 0.0001$, $n = 6$). B) Live cell binding of CA800-PR in MDA-MB-231 and MCF-7 cells. Phase contrast and NIR fluorescence images are obtained after 24 h of incubation with 100 µM of CA800-PR, respectively. C) Inhibition assay of cellular uptake of CA800-PR in MCF-7 cells cultured with serum-free medium. Cells were pre-blocked with bromsulphthalein (BSP) for 30 min and then incubated with 100 µM of CA800-PR for 1 h. Images are representative of $n = 6$ independent experiments. All fluorescence images had identical exposure times and normalization. Scale bars = 100 µm.

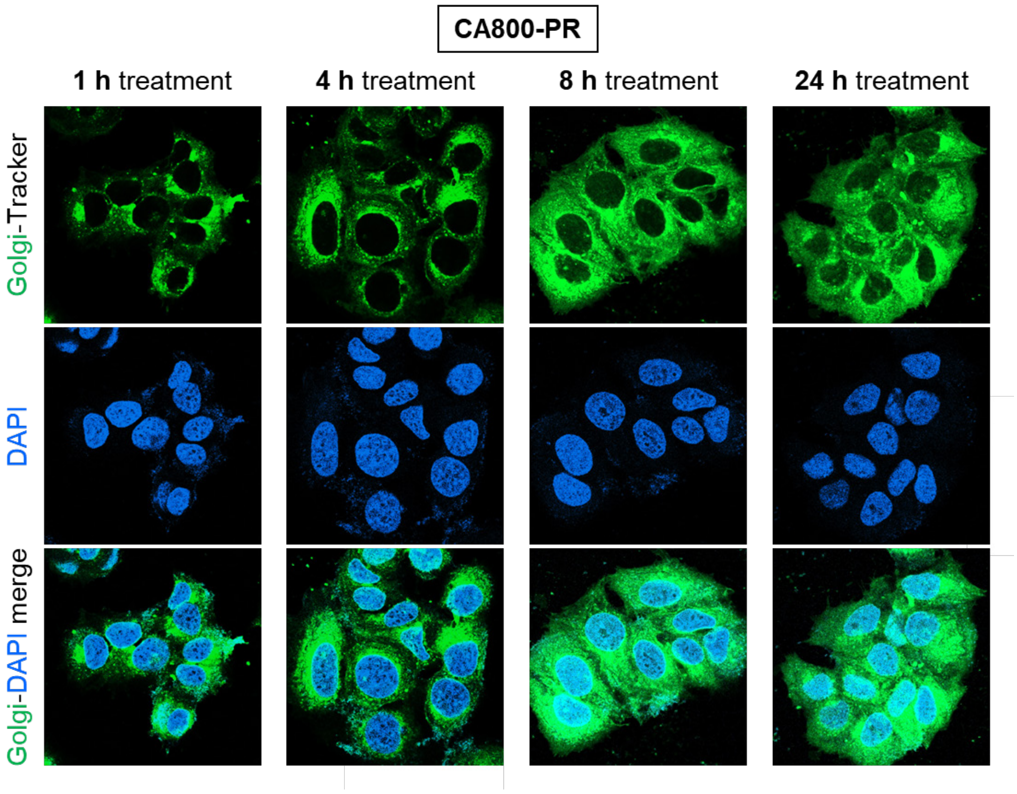

Figure S5. Confocal imaging of time-dependent Golgi dispersal by treatment of CA800-PR in MCF-7 cells. Representative images of MCF-7 cells stained with the Golgi-Tracker (green) and the nuclear marker DAPI (blue) at the indicated times after incubation with 100 µM of CA800-PR. Images are representative of $n = 6$ independent experiments. All fluorescence images had identical exposure times and normalization.

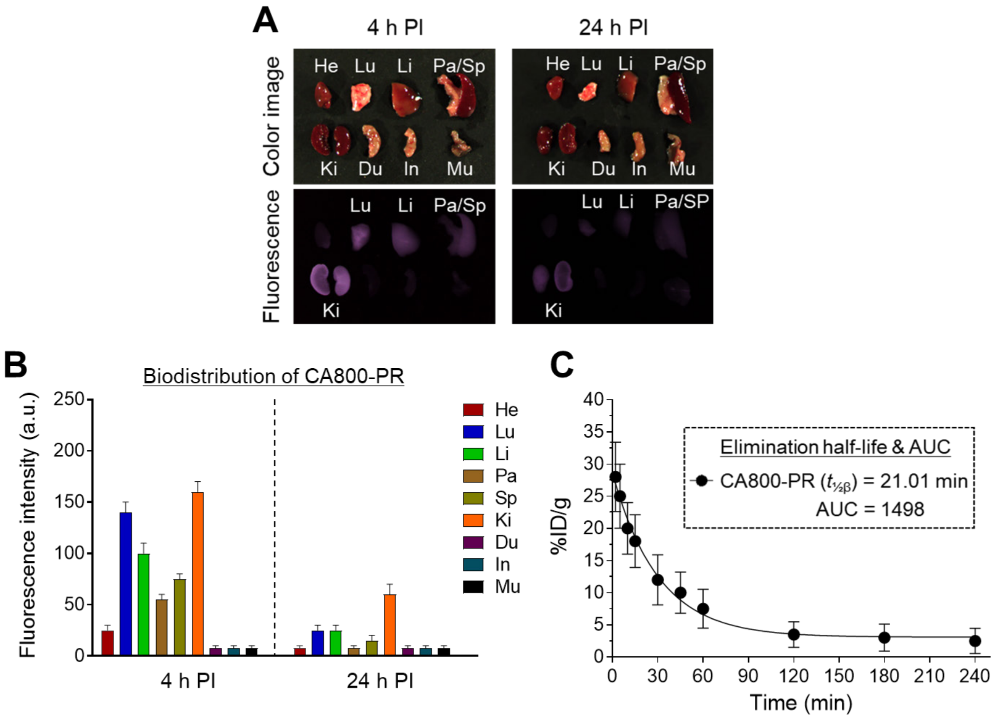

Figure S6. A) Resected major organs imaged 4 and 24 h after injection of CA800-PR. Images are representative of $n = 3$ independent experiments. All NIR fluorescence images had identical exposure times and normalization. B) Quantitative fluorescence analysis of intraoperative dissected organs imaged in a). Abbreviations: Du, duodenum; He, heart; In, intestine; Ki, kidneys; Li, liver; Lu, lungs; Mu, muscle; Pa, pancreas; Sp, spleen; and PI, post-injection. C) Pharmacokinetics of CA800-PR. Mice were intravenously injected with CA800-PR (1.5 mg kg^{-1}, $n = 3$). Blood concentration (%ID/g) decay curve, elimination half-life ($t_{1/2\beta}$), and area under the curve (AUC) values are shown.

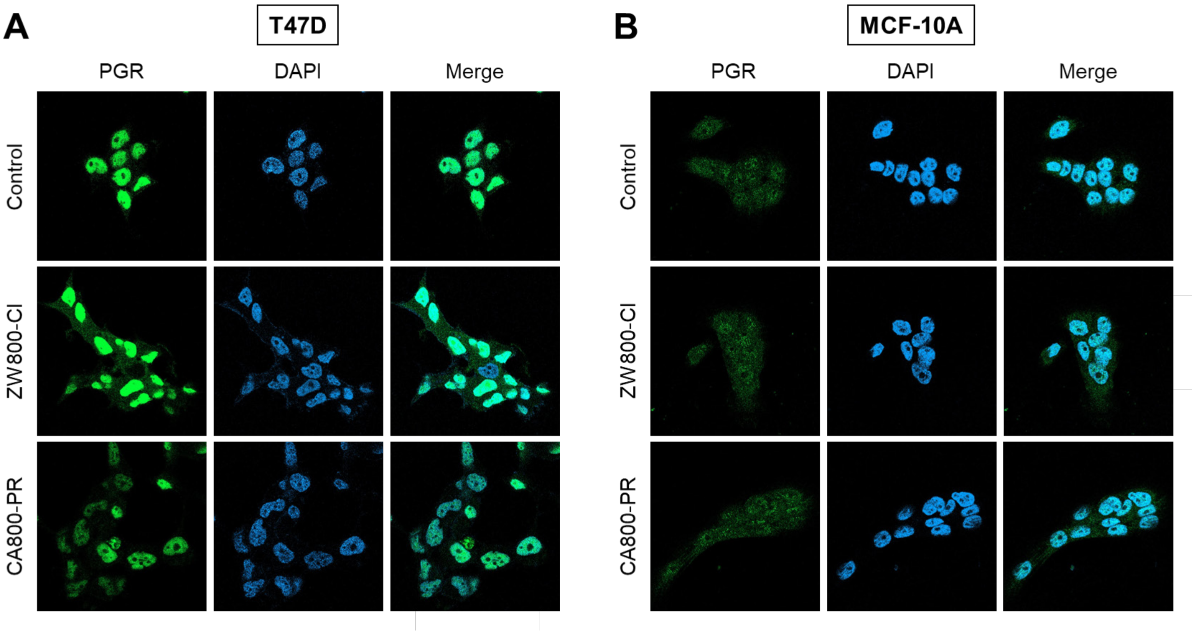

Figure S7. Quantitative analysis of PGR expression in A) T47D and B) MCF-10A cells. Representative confocal microscopy images showing endogenous PGR expression detected by immunofluorescence staining of T47D and MCF-10A cells treated with 100 µM of ZW800-Cl and CA800-PR for 24 h, respectively. Cell nuclei were stained by DAPI (blue).

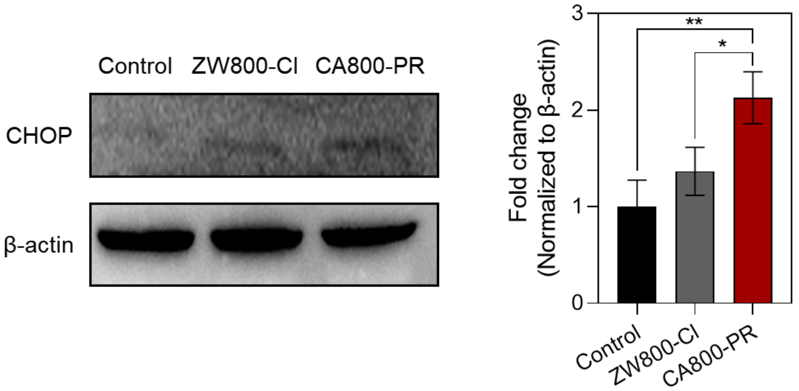

Figure S8. Representative bands and quantitative analysis of the pro-apoptotic marker (CHOP) after treatments with 100 µM of ZW800-Cl or CA800-PR for 24 h. β-actin was taken as the loading control. Data are expressed as mean ± S.D. ($^{*}p < 0.05$, $^{**}p < 0.01$, $n = 3$).

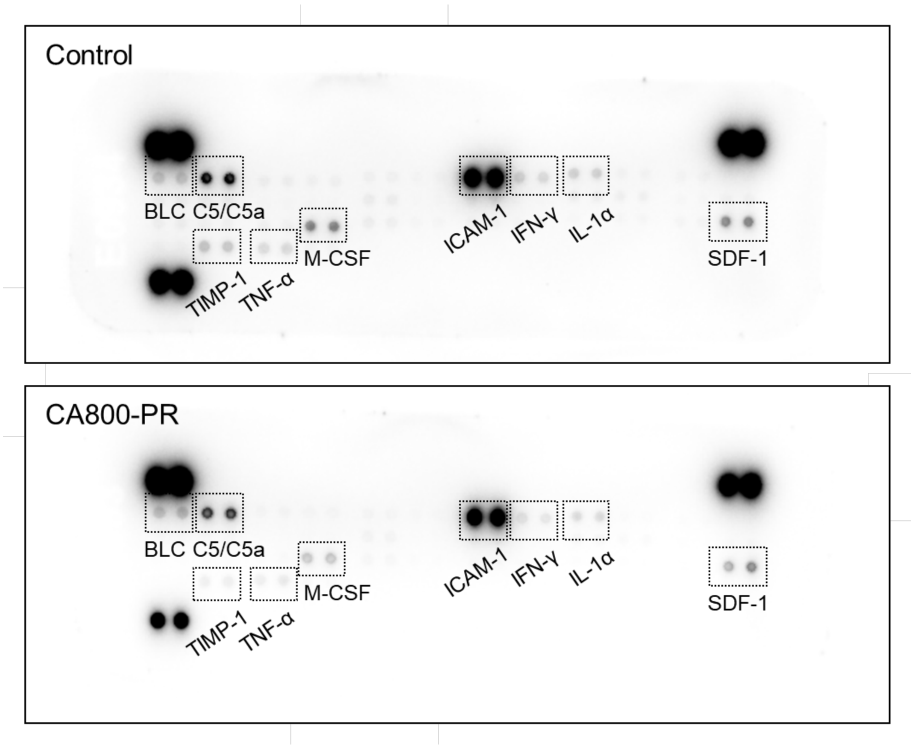

Figure S9. Cytokine profile of CA800-PR. Each cytokine was detected in duplicate. In each array, blots in the upper-left, upper-right and bottom-left represent positive controls. Target cytokines are indicated with black square dotted lines.

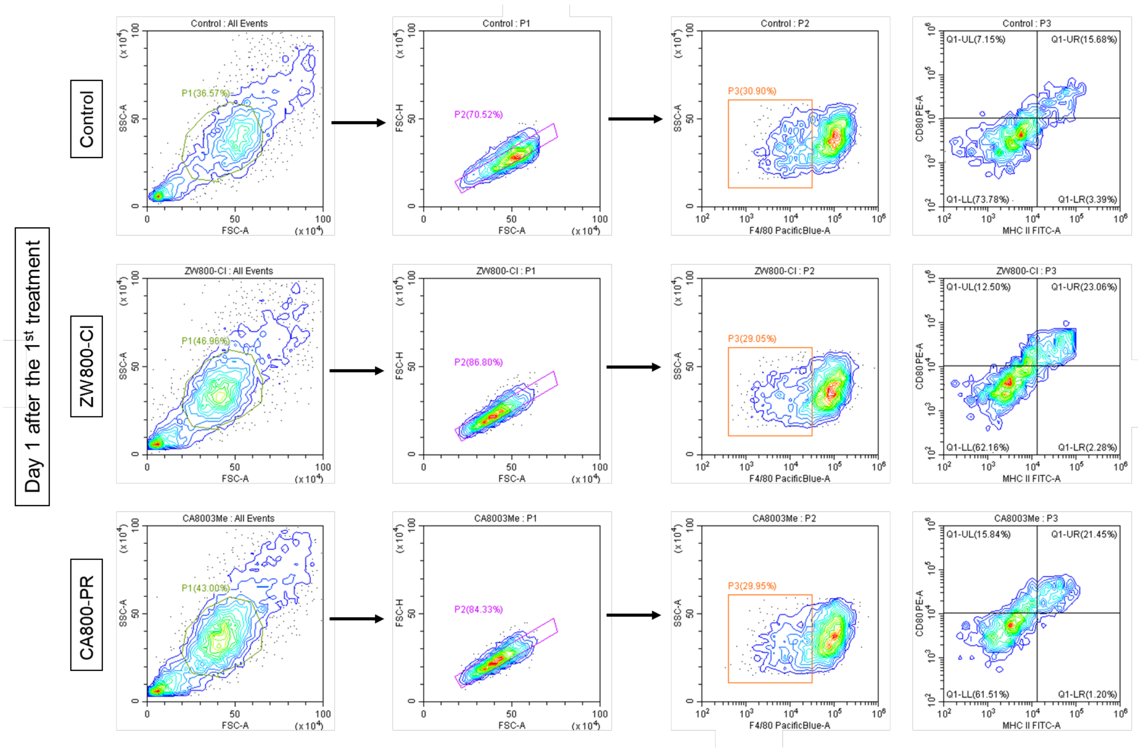

Figure S10. Representative data of the flow cytometry gating strategy applied for the assessment of DC activation after the 1st treatments with control, ZW800-Cl, or CA800-PR, respectively. BMDCs were gated, doublets and dead cells excluded. Cells were gated for expression of MHC class II$^+$ and CD80$^+$, which demonstrates the analysis of activation markers for DC maturation. Matured DCs were defined by flow cytometry, and the gating schemes are shown. FSC, forward scatter; SSC, side scatter; A, area; H, height.

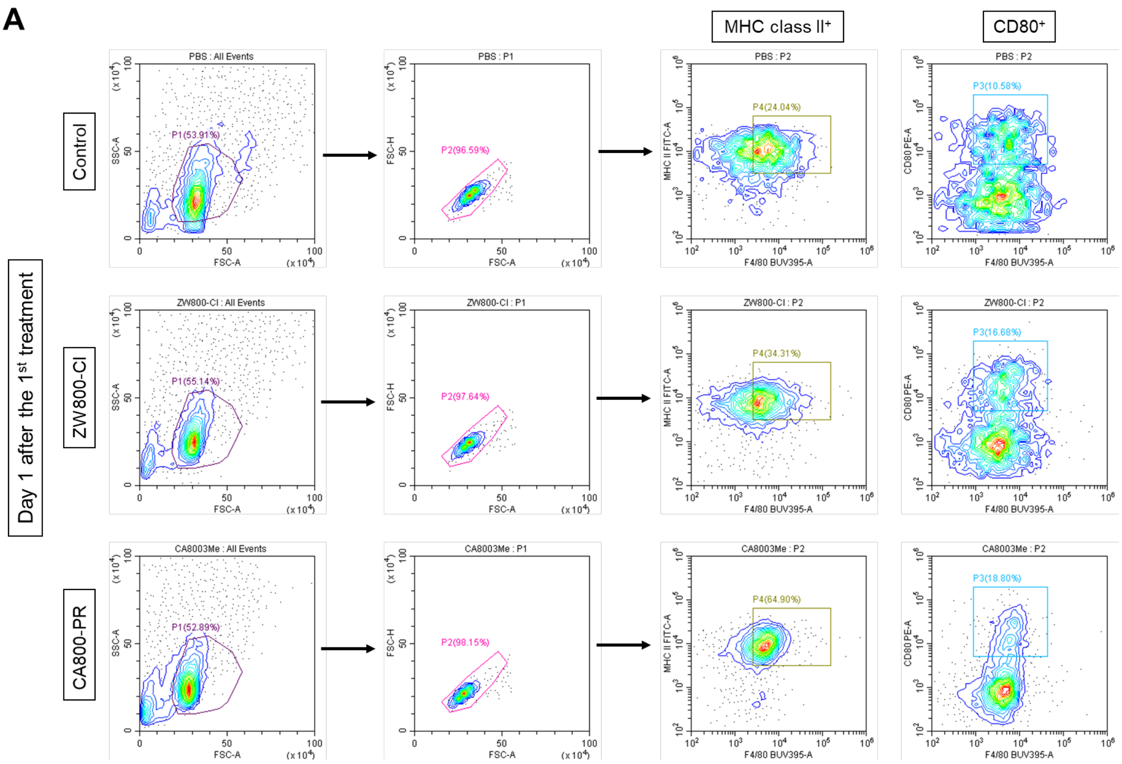

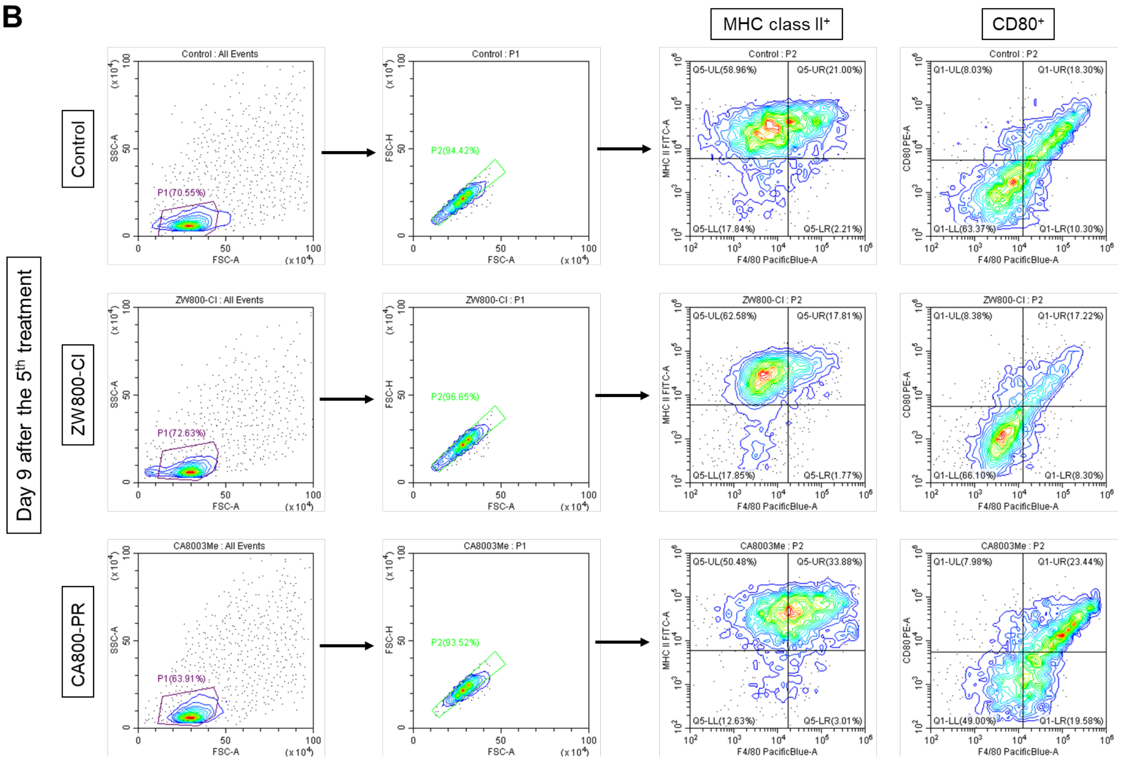

Figure S11. Flow cytometry gating strategy for identifying M1 macrophages after A) the 1st and B) the 5th treatments with control, ZW800-Cl, or CA800-PR, respectively. The activated macrophages expressing both MHC class II^{+} and CD80^{+} in spleen were defined by flow cytometry, and the gating schemes are shown. FSC, forward scatter; SSC, side scatter; A, area; H, height.

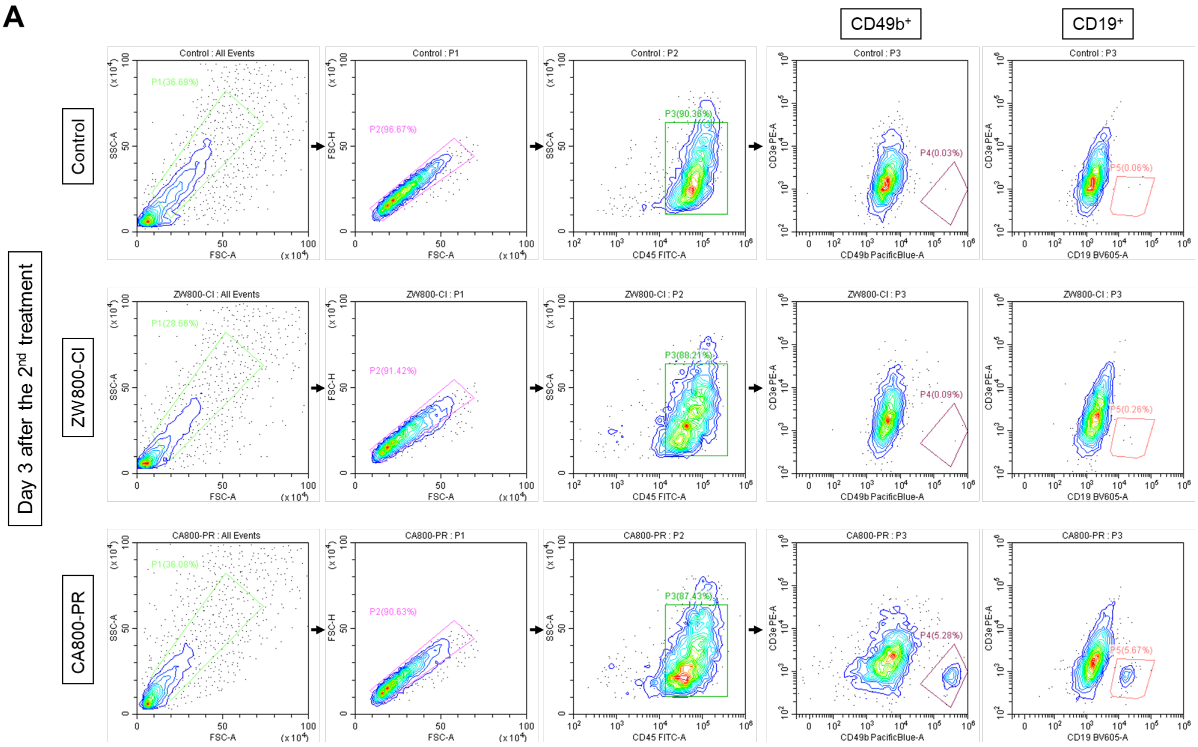

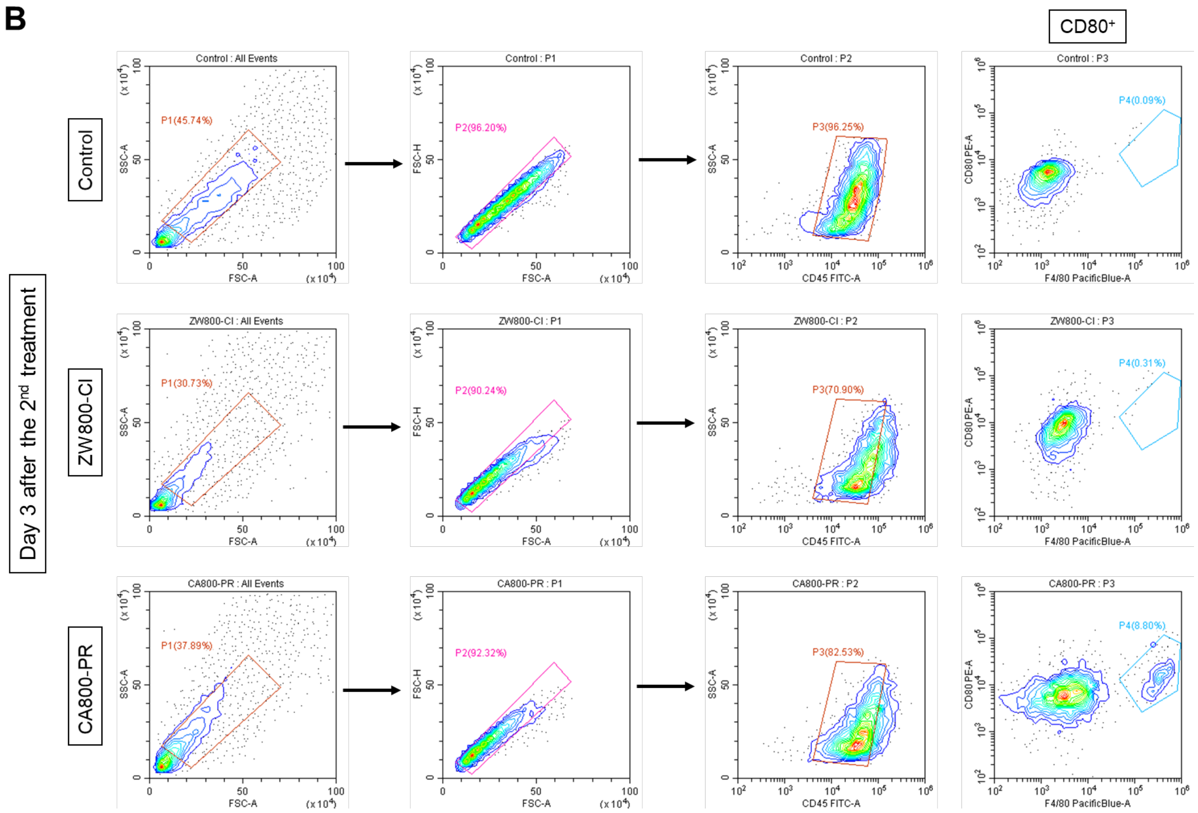

Figure S12. Flow cytometry gating strategy for identifying A) NK cells, B cells, and B) M1 macrophages after the 2nd treatment with control, ZW800-Cl, or CA800-PR, respectively. The activated NK cells, B cells, and macrophages expressing CD49b$^+$, CD19$^+$, and CD80$^+$ in the tumor were defined by flow cytometry, and the gating schemes are shown. FSC, forward scatter; SSC, side scatter; A, area; H, height.
